# Population norms for the EQ-5D-3L in China derived from the 2013 National Health Services Survey

**DOI:** 10.7189/jogh.11.08001

**Published:** 2021-02-11

**Authors:** Qiang Yao, Chaojie Liu, Yaoguang Zhang, Ling Xu

**Affiliations:** 1 School of Political Science and Public Administration, Wuhan University, Wuhan, Hubei, China; 2 School of Psychology and Public Health, La Trobe University, Melbourne, VIC, Australia; 3 Centre for Health Statistics Information, National Health Commission, Beijing, China; 4 China Health Human Resources, National Health Commission, Beijing, China

## Abstract

**Background:**

EQ-5D-3L is one of the most commonly used instruments for assessing health-related quality of life and cost-utility analyses, but it is not yet available in China. This study aims to develop population norms for the EQ-5D-3L in China in order to encourage appropriate use and interpretation of the EQ-5D-3L instrument.

**Methods:**

Data were extracted from the 2013 National Health Services Survey on a nationally representative sample of 188 720 participants. The utility index based on the 2018 Chinese preference-based value sets were calculated for the participants with different demographic and socio-economic characteristics. Differences in reported problems and visual analogue scale (VAS) and utility index scores were tested using a logistic, linear and tobit regression model, respectively.

**Results:**

The Chinese respondents were less likely to report problems on the EQ-5D dimensions compared with most populations in other countries. Pain/discomfort was the most commonly reported problem (12.6%). This resulted in a high ceiling effect (84.19%) on the utility index and high mean scores for the utility index (0.985 ± 0.056) and VAS (80.91 ± 13.74) in the Chinese population. Those who were younger, better educated, employed, married, had no illness condition, lived in a more developed region and had a higher income obtained higher scores in both VAS and utility index. The VAS and utility index scores were also associated with gender, residency and lifestyles, but not always in a consistent way. Male and rural residents had a higher VAS score but not in the utility index compared with their female and urban counterparts.

**Conclusions:**

This study provides national population norms for the EQ-5D-3L based on the 2018 Chinese preference-based value sets. The norms can be used as a reference for health evaluation studies. Cautions need to be taken for presenting and interpreting the utility index results given the high ceiling effect of the EQ-5D-3L instrument.

Health-related quality of life (HRQoL) measures the perceived well-being of people in terms of their physical, mental, social and spiritual functioning [1,2]. It adopts a philosophy of people-centred integrated care, which has attracted increasing attention around the world. HRQoL is now widely used in assessing health outcomes of both individual (eg, medical procedure) and population (eg, policy) interventions [3,4].

However, like any other health indicators, population norms are required to interpret the implications of the HRQoL measurements. They could provide a reference for tracking and comparisons [5,6], which is particularly important when there is no control group. Population norms can help define what is deemed normal or abnormal in different cultures. It is important to note that population norms may also change over time [7-9]. For HRQoL, this could be an indication of changes in population values on health or their health status or both. Therefore, the interpretation of HRQoL results has to be anchored in the historical and cultural contexts of the assessed population.

The EQ-5D, developed by the EuroQol Group in the 1990s, is one of the most commonly used instruments for assessing HRQoL. It is a simple and psychometrically sound instrument available in more than 170 languages [10]. The EQ-5D has three versions: EQ-5D-3L, EQ-5D-5L, and EQ-5D-Y. The former two were designed for adult populations and the latter one for children and adolescents aged 7 to 12 years. The EQ-5D instrument consists of a descriptive system and a self-report visual analogue scale (VAS). The descriptive system contains five items measuring the dimension of mobility, self-care, usual activities, pain/discomfort and anxiety/depression, respectively. Each dimension is assessed using either a three-point (no, moderate, severe) scale (EQ-5D-3L) or a five-point (no, slight, moderate, severe, extreme) scale (EQ-5D-5L). The combination of health states in relation to the five dimensions can be converted into a single summary index value (also known as utility index) in line with the health preference from the general population: a utility index 0 indicates death while 1 indicates full health. The VAS records self-rated general health on a vertical visual analogue scale ranging from ‘Worst imaginable health state’ (0) to ‘Best imaginable health state’ (100) [11,12]. The utility index of individuals or subpopulations needs to be examined against their population norms. More than 30 countries have subsequently established national population norms for the EQ-5D [3,4,6,7,13-26]. These norms have been successfully used in health economic evaluation and other patient-reported outcome-based studies [24].

The EuroQol Group provided population norms for the EQ-5D-3L utility index in China based on a small sample size (n = 8031) using a scoring algorithm derived from the European preference-based value sets [27,28]. However, some researchers expressed concerns about reporting the EQ-5D-3L results based on value sets derived from other countries [29]. For example, Clemens and colleagues noted the differences in the EQ-5D utility index based on the value sets derived from the preference of the local Australian population in comparison with those resulting from the UK and the US value sets [16]. Such differences may be even more profound given the greater cultural difference between China and the European countries. Indeed, Wu and colleagues identified significant differences in the utility index for some health states between the results based on the Chinese preference value sets and those derived from the UK and Japanese populations [30]. Sun and colleagues [31] reported problems in the five dimensions of the EQ-5D-3L without converting the results into utility index using the European value sets as recommended by Janssen and colleagues [27]. A large number of countries have established their own national value sets [32].

The EQ-5D-3L has been validated in a range of Chinese populations [33,34], including in the general public [31] and those with disease conditions such as hypertension[35], diabetes [36], cancer [37], and heart disease [29]. Since 2008, the EQ-5D-3L instrument has been included in the National Health Services Survey (NHSS) in China [38]. It has been recommended as a tool for conducting health technology assessment in China [29]. In 2014, Liu GG et al published the EQ-5D-3L value sets derived from a sample of urban Chinese populations [29], which prompted the development of local population norms at several provinces in China [35,39]. But Liu’s value sets suffered from a serious bias toward big cities [40]. Significant urban-rural differences in public preferences on health exist even after controlling for variations in socioeconomic status [31]. In 2018, a new version of the value sets was made available by Zhuo L et al using a national representative sample [41]. This enabled us to establish the national population norms for the EQ-5D-3L in China, which are essential for the appropriate use and interpretation of the EQ-5D instrument.

## DATA AND METHODS

### Study design and data collection

A cross-sectional survey was conducted using the EQ-5D-3L in China. Data were obtained from the 5th National Health Services Survey undertaken in September 2013 [42]. The NHSS has been the largest national representative household survey organised by the Centre for Health Statistics and Information of the Ministry of Health in China every five years since 1993. The NHHS followed a robust design and strict protocol. Data collected in the NHSS have been widely used in health services research and policy decision making [43-46]. The 5th NHSS adopted a four-stage stratified cluster random sampling strategy in selecting 93 600 households from 1560 communities/villages, covering 780 sub-districts/townships in 156 cities/counties representing the 31 provinces in mainland China. Details on the sampling procedure used in the NHSS have been reported elsewhere [43,45].

Each household member was interviewed face-to-face separately by a trained local medical worker, tapping into the demographic and socioeconomic characteristics, lifestyle and behaviors, self-reported health, and the use of health care services of the respondents. The quality of the returned questionnaire data was checked by the survey supervisors through a repeated survey on 5% of the participating households, which resulted in a 97.7% consistency. The sample was proven to be representative of the national population without age bias as indicated by the Myer’s index (2.55), DELTA dissimilarity coefficient (0.085) and GINI concentration ratio (0.0525) [42].

**Figure 1:**
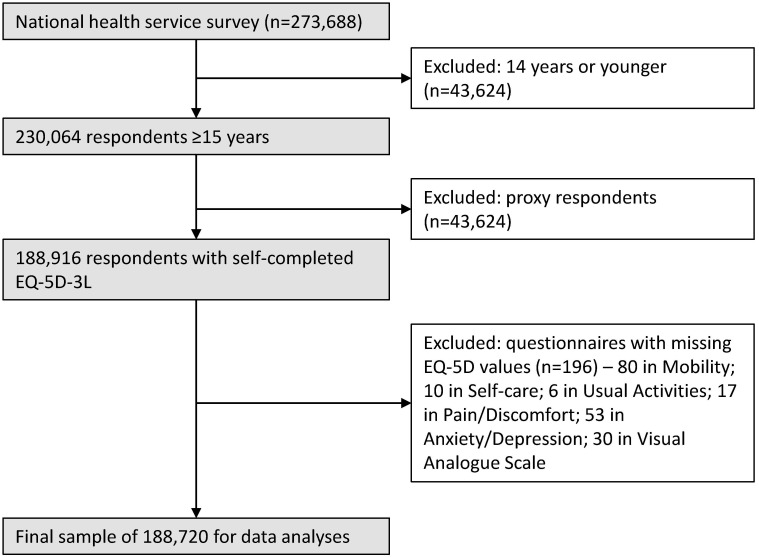
Identification of eligible participants for this study.

The EQ-5D-3L applied to those who were 15 years and older in line with other recent studies [15,35,47]. A total of 273 688 respondents completed the questionnaire, including 230 064 who were eligible for the EQ-5D-3L survey. In this study, we followed the Europe EQ-5D-3L User Guide [48] and excluded the returned questionnaires completed by a proxy respondent (a maximum of 30% of proxy responses was allowed in the NNSS for the household members who were absent at the time of the survey). We further excluded a very small number (196) of returned questionnaires containing missing values in the EQ-5D-3L. This resulted in a final sample size of 188 720 (82% of eligible participants) for data analyses (**Figure 1**).

### Measurements

We presented population norms for the EQ-5D-3L by gender and age. Given the large regional disparities in social and health development, the populations were further divided into eastern developed, western under-developed, and central regions in between, as well as urban vs rural.

The population norms were described using three indicators: percentage of reported problems on the five dimensions of the EQ-5D-3L, utility index, and VAS scores. The combinations of reported problems generated 243 possible health states. Each health state was assigned a value (utility index, ranging from 0.170 to 1.000) according to the nationally representative Chinese value sets developed by Zhuo and colleagues [41]. The Chinese value sets were derived from public preferences using the time-trade-off (TTO) technique as recommended by the EuroQol Group [41]. The VAS score is an indication of overall health perceived by an individual. The respondents were asked to rate their health along a scale ranging from 0 (worst health) to 100 (full health).

Factors associated with the EQ-5D-3L results were identified in line with the social determinants of health model proposed by the World Health Organisation [49]. Previous studies suggest that apart from age and gender, residency, education, employment, marital status, household income, illness conditions, lifestyle and behaviors are also significant predictors of HRQoL in China [31]. In this study, household income was categorised into quintiles according to the income distributions of the local cities or counties. Illness conditions were captured by reported acute conditions over the two weeks prior to the survey (yes or no), chronic conditions diagnosed by a doctor during the previous six months (yes or no), and episodes of hospital care over the previous 12 months (yes or no). Lifestyle and behaviors were measured by the current status of smoking (yes or no), drinking (yes or no during the last 12 months), and weekly physical exercise (yes or no over the last 6 months). Further details about the definitions of these variables can be found in the NHSS guidelines [42].

### Statistical analysis

We calculated the percentage of respondents reporting problems on each of the five dimensions of the EQ-5D-3L, as well as the percentage of respondents who reported problems in any dimension. Population differences in relation to the reported problems were tested using Pearson χ^2^ tests. Multivariate binary logistic regression models (with or without problems) were performed to identify the factors associated with the reported problems.

Means with standard deviations (SD) and medians with interquartile ranges (IQR) of the utility index and VAS scores of the EQ-5D-3L were calculated in line with those of previous studies [4,15,24,27,47]. Population differences in the utility index and VAS scores were tested using student *t* tests or analysis of variance (ANOVA), and Wilcoxon or Kruskal-Wallis rank sum tests. Multivariate linear regression models and Tobit regression models were performed to identify the factors associated with the VAS and the utility index scores, respectively. The Tobit approach was recommended by Zhang for censored or bounded data [35,50].

The significance level of the statistical analyses was set at 0.05. All statistical analyses were performed using STATA version 14.0 (SE) for Windows (StataCorp LLC, College Station, TX, USA). An enter approach was adopted in the regression modelling, with all of the independent variables being coded as categorical variables and compared with a reference group. Given that the statistical significance can simply be a function of large sample size, we also used the Cohen effect size (average difference in the score divided by the standard deviation of the score in the group for comparison) [51,52] to judge the significance of the differences of the utility index and VAS scores. According to Cohen, a size below 0.2 indicates a small effect, while 0.5 and 0.8 indicate a medium and a large effect size, respectively. A medium effect size (0.5) is usually considered as a difference with clinical meaning [52].

### Ethics

The present study is a secondary analysis of the NHSS 2013 data. The NHSS obtained ethics approval from the institutional review board of the Chinese National Bureau of Statistics (license number 2013-65). Informed consent was obtained from all the respondents prior to the survey. All procedures performed in the study were in accordance with the ethical standards of the Chinese National Bureau of Statistics and with the 1975 Helsinki declaration.

## RESULTS

### Characteristics of respondents

Slightly more than half of the respondents (52.4%) were women. The distribution of respondents in terms of gender, region, education, employment and marital status resembled those of the national population structure [42]. However, this study sample contained a higher proportion of respondents aged 65 years and older (17.84%) compared with the national average 11.6% [53] (**Table 1**).

**Table 1:** EQ-5D-3L VAS and utility index scores of respondents by socio-demographic characteristics

Characteristics of respondents	n	%	VAS score				Utility index				Ceiling effect (%)
**Mean**	**SD**	**t/F**	***P* value**	**Mean**	**SD**	**t/F**	***P* value**
**Demographic**											
Gender:					19.52	<0.001			5.86	<0.001	
Male	89 830	47.60	81.56	13.53			0.986	0.056			85.73
Female	98 890	52.40	80.32	13.91			0.984	0.056			82.79
Age (in years):					7030.70	<0.001			2752.71	<0.001	
15-24	14 094	7.47	90.40	8.35			0.998	0.023			97.98
25-34	24 347	12.90	88.01	9.39			0.997	0.024			96.37
35-44	35 081	18.59	84.68	11.34			0.995	0.031			92.07
45-54	41 000	21.73	81.35	12.82			0.990	0.042			87.03
55-64	40 532	21.48	77.29	13.56			0.983	0.056			79.03
65-74	22 138	11.73	73.08	14.43			0.969	0.077			69.47
75+	11 528	6.11	69.05	15.32			0.933	0.119			53.91
**Location**											
Residency:					100.06	<0.001			15.12	<0.001	
Urban	94 064	49.84	80.59	13.78			0.985	0.055			84.16
Rural	94 656	50.16	81.23	13.71			0.984	0.058			84.22
Region:					435.23	<0.001			40.53	<0.001	
Eastern	66 575	35.28	82.12	13.25			0.986	0.054			85.83
Central	58 306	30.90	80.59	13.99			0.984	0.058			83.57
Western	63 839	33.83	79.94	13.94			0.984	0.057			83.04
**Socio-economic**											
Educational attainment:					3743.86	<0.001			1542.77	<0.001	
Illiterate	22 709	12.03	73.13	15.40			0.961	0.091			67.36
Primary school	48 953	25.94	78.23	14.12			0.980	0.063			79.06
Junior middle school	65 877	34.91	83.05	12.59			0.990	0.044			88.49
Senior middle school	32 435	17.19	83.44	12.52			0.992	0.041			89.90
University/college or above	18 746	9.93	85.44	11.22			0.995	0.028			92.94
Local ranking of average household income:					712.90	<0.001			360.76	<0.001	
Lowest (<percentile 20)	35 702	18.93	77.69	15.56			0.975	0.072			76.90
Low (percentile 20-39)	35 471	18.80	80.53	13.86			0.984	0.058			83.57
Middle (percentile 40-59)	37 124	19.68	81.63	13.26			0.987	0.052			85.81
High (percentile 60-79)	39 084	20.72	81.92	12.94			0.988	0.049			86.61
Highest (≥percentile 80)	41 251	21.87	82.43	12.63			0.989	0.047			87.30
Employment:					5374.26	<0.001			2812.74	<0.001	
Employed	127 614	67.62	83.00	12.34			0.992	0.035			88.65
Retired	27 274	14.45	75.67	13.79			0.975	0.072			75.96
Student	4747	2.52	91.05	8.08			0.999	0.016			98.42
Unemployed	29 085	15.41	75.01	16.49			0.961	0.096			70.00
Marital status:					3757.16	<0.001			1623.95	<0.001	
Never married/Single	17 131	9.08	88.10	11.27			0.993	0.044			94.12
Married	155 755	82.53	80.92	13.39			0.987	0.052			85.05
Widowed	12 932	6.85	71.61	15.06			0.953	0.097			61.32
Divorced	2898	1.54	79.60	14.71			0.982	0.061			81.44
**Illness condition**											
Two-week morbidity:					-130.00	<0.001			-67.82	<0.001	
Yes	44 986	23.84	72.74	15.57			0.963	0.088			65.28
No	143 734	76.16	83.47	12.03			0.992	0.039			90.10
Chronic disease:					-160.00	<0.001			-75.39	<0.001	
Yes	50 698	26.86	72.37	15.34			0.963	0.087			65.16
No	138 022	73.14	84.05	11.63			0.993	0.036			91.18
One-year hospital admission:					-64.53	<0.001			-39.93	<0.001	
Yes	17 016	9.02	73.09	16.88			0.957	0.100			65.02
No	171 681	90.98	81.69	13.14			0.988	0.049			86.09
**Lifestyle and behaviors**											
Smoking:					13.41	<0.001			18.42	<0.001	
Yes	49 208	26.09	81.60	13.05			0.988	0.045			86.21
No	139 399	73.91	80.67	13.97			0.984	0.060			83.47
Drinking:					25.42	<0.001			31.15	<0.001	
Yes	44 005	23.32	82.28	12.46			0.991	0.037			86.95
No	144 706	76.68	80.50	14.09			0.983	0.061			83.35
Physical exercise:					0.71	0.477			25.14	<0.001	
Yes	55 843	29.67	80.94	13.10			0.989	0.038			85.10
No	132 372	70.33	80.90	14.01			0.983	0.062			83.79
**Total**	188 720	100.00	80.91	13.74	–	–	0.985	0.056	–	–	84.19

VAS – visual analogue scale

### Percentage of reported health problems

Pain/discomfort was the most frequently reported problem (12.6%), followed by problems in mobility (5.9%) and anxiety/depression (5.3%). The least reported problem was in self-care (3.1%) (**Table 2**). Women were more likely to report problems than men in relation to pain/discomfort and anxiety/depression, but less in other dimensions of the EQ-5D. The proportion of respondents reporting problems increased with age. Socioeconomic gradients were evident, and those with a higher socio-economic status (better educated, higher income and employed) were less likely to report problems. Rural residents and those residing in the less developed central and western regions were more likely to report problems on all of the five dimensions than others. Those who did not smoke or drink reported more problems than those who did (**Table 3**).

**Table 2:** Percentage (%) of respondents reporting problems in the five dimensions of EQ-5D-3L

Variable	Any problems	Mobility			Self-care			Usual activities			Pain/discomfort			Anxiety/depression		
**No**	**Some**	**Bed confined**	**No**	**Some**	**Unable**	**No**	**Some**	**Unable**	**No**	**Some**	**Extreme**	**No**	**Some**	**Extreme**
**Gender**	*P* < 0.001	*P* = 0.008			*P* = 0.029			*P* < 0.001			*P* < 0.001			*P* < 0.001		
Male	14.3	94.3	5.4	0.3	97.0	2.5	0.5	95.5	3.7	0.9	89.0	10.5	0.5	95.4	4.4	0.3
Female	17.2	94.0	5.7	0.3	96.9	2.7	0.4	95.2	4.0	0.8	85.9	13.5	0.6	94.1	5.6	0.3
**Age (in years)**	*P* < 0.001	*P* < 0.001			*P* < 0.001			*P* < 0.001			*P* < 0.001			*P* < 0.001		
15-24	2.0	99.5	0.4	0.1	99.7	0.3	0.1	99.5	0.3	0.1	98.8	1.1	0.1	99.1	0.8	0.1
25-34	3.6	99.3	0.6	0.1	99.6	0.4	0.1	99.4	0.5	0.1	97.8	2.1	0.1	98.4	1.5	0.1
35-44	7.9	98.5	1.4	0.1	99.2	1.3	0.2	98.7	1.0	0.2	94.3	5.6	0.2	96.6	3.2	0.2
45-54	13.0	96.9	3.0	0.2	98.5	2.6	0.4	97.5	2.1	0.4	89.5	10.1	0.4	95.3	4.4	0.2
55-64	21.0	93.6	6.2	0.3	97.0	5.9	0.8	95.2	4.0	0.8	82.4	17.0	0.7	93.1	6.6	0.3
65-74	30.5	86.6	12.8	0.6	93.2	14.4	2.8	89.6	8.8	1.6	74.7	24.3	1.0	91.0	8.6	0.4
75+	46.1	70.2	27.7	2.2	82.8	2.6	0.4	75.4	19.7	4.9	64.8	33.2	2.0	86.5	12.7	0.8
**Residency**	*P* = 0.698	*P* = 0.008			*P* < 0.001			*P* < 0.001			*P* = 0.107			*P* < 0.001		
Urban	15.8	94.3	5.4	0.3	97.2	2.4	0.4	95.7	3.5	0.8	87.6	12.0	0.5	95.0	4.7	0.3
Rural	15.8	94.0	5.7	0.3	96.7	2.9	0.4	95.0	4.2	0.8	87.2	12.3	0.5	94.4	5.3	0.3
**Region**	*P* < 0.001	*P* < 0.001			*P* < 0.001			*P* < 0.001			*P* < 0.001			*P* < 0.001		
Eastern	14.2	94.6	5.1	0.3	97.2	2.4	0.4	95.8	3.4	0.8	88.9	10.7	0.4	95.8	4.0	0.2
Central	16.4	94.0	5.7	0.3	96.9	2.6	0.5	95.4	3.8	0.8	86.6	12.8	0.6	94.5	5.3	0.3
Western	17.0	93.8	5.9	0.3	96.8	2.9	0.4	94.9	4.3	0.8	86.5	12.9	0.6	93.8	5.8	0.4
**Educational attainment**	*P* < 0.001	*P* < 0.001			*P* < 0.001			*P* < 0.001			*P* < 0.001			*P* < 0.001		
Illiterate	32.6	84.6	14.5	1.0	91.1	7.6	1.3	86.6	10.9	2.4	73.4	25.3	1.3	88.3	11.1	0.6
Primary school	20.9	92.0	7.6	0.4	96.0	3.5	0.5	93.9	5.1	1.0	83.0	16.4	0.7	93.4	6.3	0.3
Junior middle school	11.5	96.4	3.4	0.2	98.2	1.5	0.3	97.3	2.3	0.5	91.0	8.7	0.3	96.1	3.7	0.2
Senior middle school	10.1	97.1	2.7	0.2	98.6	1.2	0.2	97.8	1.8	0.4	92.1	7.6	0.3	96.8	3.0	0.2
University/college or above	7.1	98.3	1.6	0.1	99.2	0.7	0.1	98.8	1.0	0.2	95.0	4.8	0.1	97.5	2.4	0.1
**Local ranking of average household income**	*P* < 0.001	*P* < 0.001			*P* < 0.001			*P* < 0.001			*P* < 0.001			*P* < 0.001		
Lowest (<percentile 20)	23.1	90.2	9.3	0.5	94.7	4.6	0.7	91.9	6.6	1.5	81.7	17.4	0.9	91.6	7.9	0.5
Low (percentile 20-39)	16.4	93.9	5.7	0.4	96.9	2.7	0.5	95.2	3.9	0.9	87.0	12.4	0.5	94.4	5.4	0.2
Middle (percentile 40-59)	14.2	95.1	4.7	0.3	97.4	2.2	0.4	96.2	3.2	0.7	88.3	11.2	0.5	95.4	4.4	0.2
High (percentile 60-79)	13.4	95.4	4.3	0.3	97.7	2.0	0.3	96.5	3.0	0.6	89.3	10.3	0.4	95.8	4.0	0.2
Highest (≥percentile 80)	12.7	95.7	4.0	0.2	97.8	1.9	0.3	96.7	2.7	0.6	90.0	9.7	0.3	96.2	3.7	0.2
**Employment**	*P* < 0.001	*P* < 0.001			*P* < 0.001			*P* < 0.001			*P* < 0.001			*P* < 0.001		
Employed	11.4	97.1	2.9	0.1	98.7	1.2	0.1	97.8	2.0	0.2	90.9	8.9	0.3	96.0	3.9	0.2
Retired	24.0	89.7	9.7	0.6	94.8	4.4	0.9	92.3	6.1	1.5	80.9	18.3	0.8	94.0	5.7	0.3
Student	1.6	99.6	0.3	0.0	99.8	0.2	0.0	99.8	0.2	0.0	99.2	0.8	0.1	99.3	0.6	0.0
Unemployed	30.0	84.7	14.2	1.2	91.0	7.5	1.5	86.8	10.4	2.8	76.1	22.4	1.5	89.1	10.1	0.8
**Marital status**	*P* < 0.001	*P* < 0.001			*P* < 0.001			*P* < 0.001			*P* < 0.001			*P* < 0.001		
Never married/Single	5.9	97.7	2.1	0.2	98.5	1.2	0.3	97.8	1.7	0.5	96.3	3.5	0.3	97.4	2.4	0.2
Married	15.0	95.0	4.8	0.3	97.5	2.2	0.4	96.1	3.2	0.7	87.9	11.6	0.5	95.1	4.7	0.2
Divorced	38.7	79.7	19.1	1.1	88.5	10.0	1.5	83.4	13.7	2.8	69.3	29.4	1.3	87.4	12.0	0.7
Widowed	18.6	94.0	5.7	0.4	96.7	2.9	0.4	95.0	4.1	0.9	86.2	13.1	0.7	91.6	7.7	0.8
**Two-week morbidity**	*P* < 0.001	*P* < 0.001			*P* < 0.001			*P* < 0.001			*P* < 0.001			*P* < 0.001		
Yes	34.7	85.6	13.5	0.9	92.4	6.4	1.2	88.4	9.3	2.3	70.7	27.9	1.5	88.3	11.1	0.7
No	9.9	96.8	3.0	0.2	98.4	1.5	0.2	97.5	2.1	0.3	92.6	7.2	0.2	96.8	3.1	0.1
**Chronic disease**	*P* < 0.001	*P* < 0.001			*P* < 0.001			*P* < 0.001			*P* < 0.001			*P* < 0.001		
Yes	34.8	85.4	13.8	0.8	92.3	6.5	1.2	88.2	9.6	2.2	70.9	27.6	1.4	88.3	11.1	0.6
No	8.8	97.4	2.5	0.1	98.6	1.2	0.2	98.0	1.7	0.3	93.4	6.4	0.2	97.1	2.8	0.1
**One-year hospital admission**	*P* < 0.001	*P* < 0.001			*P* < 0.001			*P* < 0.001			*P* < 0.001			*P* < 0.001		
Yes	35.0	83.4	15.3	1.3	90.6	7.7	1.7	86.0	11.0	3.1	70.8	27.2	2.1	87.0	12.2	0.9
No	13.9	95.2	4.6	0.2	97.6	2.1	0.3	96.3	3.1	0.6	89.0	10.6	0.4	95.5	4.3	0.2
**Smoking**	*P* < 0.001	*P* < 0.001			*P* < 0.001			*P* < 0.001			*P* < 0.001			*P* < 0.001		
Yes	13.8	95.3	4.5	0.2	97.8	2.0	0.2	96.5	3.0	0.5	89.2	10.5	0.4	95.5	4.3	0.2
No	16.5	93.7	5.9	0.4	96.6	2.9	0.5	95.0	4.1	0.9	86.8	12.7	0.6	94.5	5.3	0.3
**Drinking**	*P* < 0.001	*P* < 0.001			*P* < 0.001			*P* < 0.001			*P* < 0.001			*P* < 0.001		
Yes	13.1	96.2	3.7	0.1	98.5	1.4	0.1	97.5	2.3	0.3	89.7	10.0	0.2	96.0	3.9	0.2
No	16.7	93.5	6.1	0.4	96.5	3.0	0.5	94.7	4.3	1.0	86.7	12.7	0.6	94.4	5.4	0.3
**Physical exercise**	*P* < 0.001	*P* < 0.001			*P* < 0.001			*P* < 0.001			*P* < 0.001			*P* < 0.001		
Yes	14.9	95.5	4.4	0.1	98.1	1.8	0.1	96.8	2.9	0.3	88.1	11.6	0.3	95.7	4.1	0.1
No	16.2	93.6	6.0	0.4	96.5	3.0	0.6	94.8	4.2	1.0	87.1	12.3	0.6	94.3	5.4	0.3
**Total**	15.8	94.1	5.5	0.3	97.0	2.6	0.4	95.4	3.8	0.8	87.4	12.1	0.5	94.7	5.0	0.3

**Table 3:** Factors associated with the percentage of problems reported by respondents in the five EQ-5D dimensions – results of logistic regression models

Variable	Mobility			Self-care			Usual activities			Pain/discomfort			Anxiety/depression		
AOR*	**95% CI**	**P**	**AOR**	**95% CI**	**P**	**AOR**	**(95% CI**	**P**	**AOR**	**(95% CI)**	**P**	**AOR**	**(95% CI)**	**P**
**Gender**															
Male (reference)															
Female	0.727	0.688, 0.768	<0.001	0.615	0.573, 0.661	<0.001	0.652	0.613, 0.692	<0.001	1.196	1.149, 1.245	<0.001	1.064	1.006, 1.124	0.030
**Age in years**															
15-24 (reference)															
25-34	1.818	1.308, 2.525	<0.001	1.806	1.233, 2.646	0.002	1.839	1.333, 2.537	<0.001	1.717	1.417, 2.082	<0.001	2.225	1.771, 2.795	<0.001
35-44	3.754	2.749, 5.126	<0.001	3.413	2.378, 4.898	<0.001	3.567	2.627, 4.842	<0.001	3.958	3.299, 4.750	<0.001	4.505	3.611, 5.621	<0.001
45-54	6.582	4.847, 8.939	<0.001	5.124	3.602, 7.287	<0.001	5.699	4.221, 7.693	<0.001	6.296	5.253, 7.546	<0.001	5.315	4.261, 6.631	<0.001
55-64	9.276	6.829, 12.601	<0.001	6.454	4.535, 9.187	<0.001	7.042	5.211, 9.515	<0.001	8.369	6.973, 10.045	<0.001	5.947	4.758, 7.435	<0.001
65-74	14.618	10.734, 19.908	<0.001	9.737	6.820, 13.903	<0.001	10.863	8.013, 14.725	<0.001	10.229	8.498, 12.313	<0.001	5.905	4.700, 7.418	<0.001
75+	31.140	22.803, 42.526	<0.001	19.503	13.616, 27.936	<0.001	22.489	16.541, 30.577	<0.001	13.617	11.262, 16.465	<0.001	7.359	5.823, 9.300	<0.001
**Location**															
Urban (reference)															
Rural	1.068	1.015, 1.123	0.011	1.186	1.109, 1.267	<0.001	1.165	1.102, 1.233	<0.001	1.000	0.965, 1.036	0.993	1.037	0.988, 1.088	0.136
**Region**															
Eastern (reference)															
Central	1.213	1.149, 1.280	<0.001	1.205	1.122, 1.294	<0.001	1.178	1.109, 1.252	<0.001	1.320	1.271, 1.371	<0.001	1.375	1.302, 1.453	<0.001
Western	1.444	1.369, 1.522	<0.001	1.423	1.326, 1.526	<0.001	1.503	1.417, 1.594	<0.001	1.492	1.437, 1.549	<0.001	1.670	1.584, 1.761	<0.001
**Educational attainment**															
Illiterate (reference)															
Primary school	0.827	0.780, 0.877	<0.001	0.750	0.695, 0.809	<0.001	0.721	0.677, 0.769	<0.001	0.859	0.822, 0.897	<0.001	0.775	0.730, 0.822	<0.001
Junior middle school	0.716	0.667, 0.768	<0.001	0.655	0.597, 0.719	<0.001	0.625	0.578, 0.676	<0.001	0.700	0.665, 0.736	<0.001	0.704	0.657, 0.754	<0.001
Senior middle school	0.592	0.540, 0.649	<0.001	0.540	0.477, 0.611	<0.001	0.522	0.471, 0.578	<0.001	0.652	0.612, 0.694	<0.001	0.669	0.612, 0.730	<0.001
University/college or above	0.493	0.429, 0.565	<0.001	0.474	0.393, 0.572	<0.001	0.438	0.375, 0.512	<0.001	0.602	0.551, 0.657	<0.001	0.740	0.657, 0.833	<0.001
**Local ranking of average household income**															
Lowest < percentile 20 (reference)															
Low percentile 20-39	0.803	0.753, 0.855	<0.001	0.819	0.754, 0.891	<0.001	0.789	0.735, 0.846	<0.001	0.802	0.766, 0.840	<0.001	0.791	0.744, 0.842	<0.001
Middle percentile 40-59	0.703	0.658, 0.752	<0.001	0.732	0.671, 0.799	<0.001	0.690	0.641, 0.743	<0.001	0.758	0.723, 0.794	<0.001	0.684	0.641, 0.730	<0.001
High percentile 60-79	0.651	0.608, 0.696	<0.001	0.669	0.612, 0.732	<0.001	0.637	0.591, 0.686	<0.001	0.679	0.647, 0.712	<0.001	0.623	0.584, 0.666	<0.001
Highest ≥ percentile 80	0.627	0.585, 0.671	<0.001	0.654	0.596, 0.717	<0.001	0.622	0.576, 0.671	<0.001	0.647	0.616, 0.679	<0.001	0.580	0.541, 0.621	<0.001
**Employment**															
Employed (reference)															
Retired	1.884	1.748, 2.031	<0.001	2.522	2.278, 2.793	<0.001	2.132	1.959, 2.321	<0.001	1.072	1.017, 1.130	0.010	0.981	0.907, 1.061	0.634
Student	0.806	0.457, 1.421	0.456	0.884	0.458, 1.705	0.713	0.591	0.313, 1.116	0.105	0.639	0.450, 0.907	0.012	0.706	0.479, 1.042	<0.001
Unemployed	2.670	2.521, 2.826	<0.001	3.345	3.096, 3.614	<0.001	3.097	2.907, 3.299	<0.001	1.571	1.508, 1.637	<0.001	1.675	1.584, 1.771	<0.001
**Marital status:**															
Never married/Single (reference)															
Married	0.489	0.430, 0.556	<0.001	0.426	0.365, 0.497	<0.001	0.417	0.365, 0.476	<0.001	0.798	0.725, 0.879	<0.001	0.546	0.487, 0.612	<0.001
Divorced	0.608	0.528, 0.699	<0.001	0.547	0.462, 0.648	<0.001	0.516	0.446, 0.597	<0.001	0.927	0.833, 1.033	0.170	0.676	0.593, 0.772	<0.001
Widowed	0.814	0.660, 1.005	0.055	0.744	0.570, 0.971	<0.001	0.729	0.582, 0.914	0.006	1.158	0.997, 1.345	0.055	1.088	0.911, 1.300	0.350
**Two-week morbidity**															
Yes (reference)															
No	0.606	0.568, 0.646	<0.001	0.595	0.545, 0.649	<0.001	0.586	0.545, 0.630	<0.001	0.496	0.473, 0.519	<0.001	0.562	0.527, 0.600	<0.001
**Chronic disease**															
Yes (reference)															
No	0.489	0.458, 0.523	<0.001	0.552	0.503, 0.605	<0.001	0.488	0.452, 0.526	<0.001	0.488	0.465, 0.512	<0.001	0.503	0.47, 0.538	<0.001
**One-year hospital admission**															
Yes (reference)															
No	0.468	0.443, 0.494	<0.001	0.459	0.428, 0.492	<0.001	0.440	0.414, 0.467	<0.001	0.520	0.499, 0.543	<0.001	0.525	0.497, 0.555	<0.001
**Smoking**															
Yes (reference)															
No	1.188	1.118, 1.263	<0.001	1.323	1.219, 1.437	<0.001	1.279	1.194, 1.370	<0.001	1.021	0.977, 1.066	0.360	1.055	0.992, 1.122	<0.001
**Drinking**															
Yes (reference)															
No	1.338	1.254, 1.427	<0.001	1.885	1.716, 2.069	<0.001	1.649	1.529, 1.778	<0.001	0.987	0.945, 1.031	0.565	1.088	1.022, 1.158	<0.001
**Physical exercise**															
Yes (reference)															
No	2.091	1.970, 2.219	<0.001	2.549	2.348, 2.767	<0.001	2.325	2.172, 2.489	<0.001	1.299	1.249, 1.352	<0.001	1.424	1.345, 1.508	<0.001

AOR – adjusted odds ratio, CI – 95% confidence interval

### EQ-5D-3L utility index and VAS score

**Figure 2:**
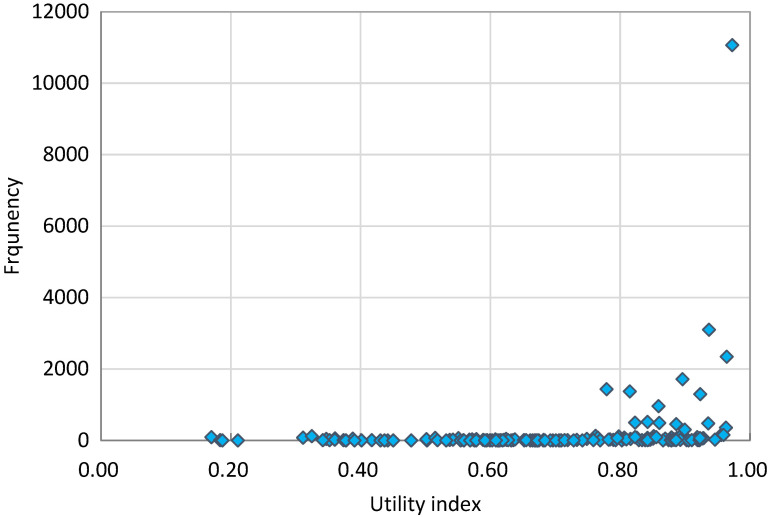
Distribution of EQ-5D-3L utility index scores in the respondents with a health problem.

The distribution of the utility index was negatively skewed (**Figure 2**). The respondents reported 161 health states out of a possible 243. More than 84% of respondents had a full health state (“11111”), followed by the states of “11121” (5.87%), “11122” (1.64%), “11112” (1.24%), “21121” (0.91%), “22222” (0.76%), “22221” (0.73%), and “21111” (0.68%). This generated a very high mean score of the utility index: 0.985 (SD = 0.056). Details about the distributional information of the 161 EQ-5D-3L health states, and the median and IQR of utility index can be found in the supplementary file (Table S1 and S2 in **the** **Online Supplementary Document**).

**Figure 3:**
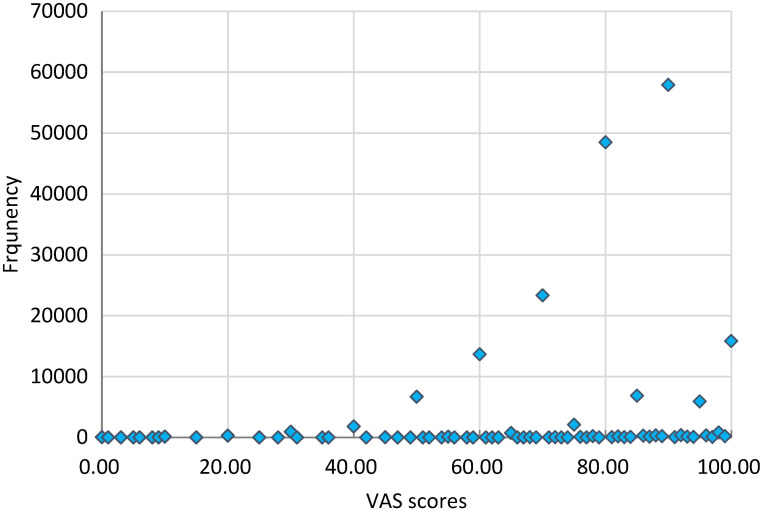
Distribution of EQ-5D-3L VAS scores in the respondents. (Mean = 80.91, SD = 13.74, Median = 80.00, IQR = 70.00-90.00).

**Figure 4:**
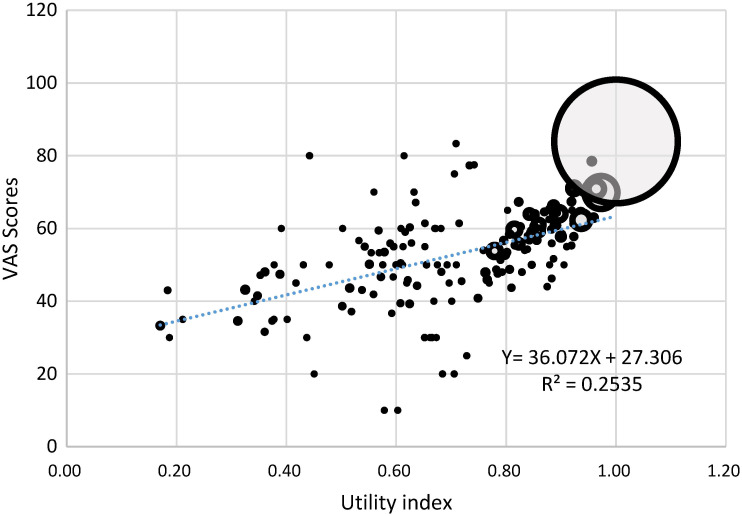
Correlation between EQ-5D-3L VAS scores and utility index (r = 0.4537, *P* < 0.05). Note: The size of the bubbles represents the frequency of individuals with a corresponding combination of the two scores

Similarly, the distribution of the VAS score was also negatively skewed (**Figure 3**). More than 40% of respondents reported a higher than 90 VAS score. But the mean and median values of VAS were similar: 80.91 (SD = 13.74) vs 80.00 (IQR: 70.00 - 90.00). Details about the median and IQR of VAS score can be found in the supplementary file (Table S2 in the **Online Supplementary Document**). The VAS scores were moderately correlated with the utility index scores (r = 0.4537, *P* < 0.05) in the total sample (**Figure 4**), as well as in the subsamples stratified by gender and age (Figure S1 to S9 in the **Online Supplementary Document**).

Men and rural residents had higher VAS scores (but not in the utility index) than women and urban residents. Younger respondents had higher VAS and utility index scores than their older counterparts. Socioeconomic gradients were evident, and those with a higher socio-economic status (eg, better educated, higher income, employed and living in developed areas) had higher utility index and VAS scores. Those who did not smoke or drink had a lower utility index, but not in VAS scores (**Table 1** and **Table 4**). In addition, the effect size of differences in age, region, education attainment, income level, employment status, marital status, two-week morbidity, chronic disease, hospitalisation, and exercising on the utility index exceeded the medium effect size of 0.5 (**Table 4**). The median and IQR results also supported these findings (Table S2 in the **Online Supplementary Document****).**

**Table 4:** Factors associated with VAS and utility index scores – results of multiple linear and Tobit regression analyses

Variable	EQ-5D VAS					EQ-5D utility				
β*	**SE**	**95% CI**	**P**	**Effect size**	**β**	**SE**	**95% CI**	***P* value**	**Effect size**
**Gender**										
Male (reference)										
Female	-0.163	0.072	-0.304, -0.023	0.023	-0.01	0.003	0.002	-0.001, 0.006	0.114	0.05
**Age**										
15-24 (reference)										
25-34	-2.406	0.120	-2.642, -2.171	<0.001	-0.18	-0.051	0.006	-0.063, -0.039	<0.001	-0.91
35-44	-5.171	0.128	-5.422, -4.920	<0.001	-0.38	-0.105	0.006	-0.116, -0.093	<0.001	-1.87
45-54	-7.548	0.131	-7.804, -7.292	<0.001	-0.55	-0.136	0.006	-0.147, -0.124	<0.001	-2.42
55-64	-9.507	0.140	-9.782, -9.233	<0.001	-0.69	-0.156	0.006	-0.168, -0.145	<0.001	-2.79
65-74	-11.709	0.165	-12.033, -11.385	<0.001	-0.85	-0.180	0.006	-0.192, -0.168	<0.001	-3.21
75+	-13.949	0.208	-14.355, -13.542	<0.001	-1.02	-0.236	0.006	-0.248, -0.223	<0.001	-4.20
**Location**										
Urban (reference)										
Rural	0.731	0.062	0.609, 0.854	<0.001	0.05	-0.002	0.001	-0.005, 0.001)	0.195	-0.03
**Region**										
Eastern (reference)										
Central	-1.439	0.067	-1.570, -1.309	<0.001	-0.10	-0.019	0.002	-0.022, -0.016	<0.001	-0.34
Western	-2.715	0.065	-2.843, -2.588	<0.001	-0.20	-0.031	0.002	-0.034, -0.028	<0.001	-0.55
**Educational attainment**										
Illiterate (reference)										
Primary school	1.964	0.113	1.743, 2.186	<0.001	0.14	0.020	0.002	0.016, 0.023	<0.001	0.35
Junior middle school	3.151	0.116	2.925, 3.378	<0.001	0.23	0.036	0.002	0.032, 0.04	<0.001	0.65
Senior middle school	2.996	0.129	2.744, 3.249	<0.001	0.22	0.043	0.003	0.038, 0.048	<0.001	0.76
University/college or above	2.754	0.142	2.475, 3.033	<0.001	0.20	0.041	0.003	0.035, 0.048	<0.001	0.74
**Local ranking of average household income**										
Lowest (<percentile 20) (reference)										
Low (percentile 20-39)	1.371	0.095	1.185, 1.556	<0.001	0.10	0.021	0.002	0.017, 0.024	<0.001	0.37
Middle (percentile 40-59)	2.042	0.092	1.861, 2.223	<0.001	0.15	0.031	0.002	0.027, 0.034	<0.001	0.54
High (percentile 60-79)	2.376	0.091	2.196, 2.555	<0.001	0.17	0.038	0.002	0.034, 0.041	<0.001	0.67
Highest (≥percentile 80)	2.841	0.091	2.662, 3.021	<0.001	0.21	0.041	0.002	0.037, 0.044	<0.001	0.72
**Employment**										
Employed (reference)										
Retired	-0.846	0.112	-1.066, -0.627	<0.001	-0.06	-0.022	0.002	-0.027, -0.018	<0.001	-0.40
Student	0.430	0.151	0.134, 0.727	0.004	0.03	0.017	0.009	-0.002, 0.035	<0.001	0.29
Unemployed	-2.942	0.097	-3.132, -2.753	<0.001	-0.21	-0.063	0.002	-0.066, -0.059	<0.001	-1.11
**Marital status**										
Never married/Single (reference)										
Married	0.687	0.120	0.452, 0.923	<0.001	0.05	0.042	0.004	0.035, 0.050	<0.001	0.75
Divorced	-0.315	0.184	-0.676, 0.046	0.087	-0.02	0.023	0.004	0.015, 0.032	<0.001	0.42
Widowed	-1.367	0.267	-1.89, -0.843	<0.001	-0.10	-0.001	0.006	-0.013, 0.01	0.849	-0.02
**Two-week morbidity**										
Yes (reference)										
No	3.282	0.105	3.076, 3.487	<0.001	0.24	0.059	0.002	0.055, 0.063	<0.001	1.05
**Chronic disease**										
Yes (reference)										
No	4.756	0.103	4.554, 4.958	<0.001	0.35	0.063	0.002	0.059, 0.066	<0.001	1.12
**One-year hospital admission**										
Yes (reference)										
No	4.049	0.119	3.816, 4.282	0.281	0.29	0.066	0.002	0.062, 0.070	<0.001	1.18
**Smoking**										
Yes (reference)										
No	-0.083	0.077	-0.234, 0.068	<0.001	-0.01	-0.009	0.002	-0.012, -0.006	<0.001	-0.16
**Drinking**										
Yes (reference)										
No	-0.799	0.074	-0.944, -0.654	<0.001	-0.06	-0.007	0.002	-0.011, -0.004	<0.001	-0.13
**Physical exercise**										
Yes (reference)										
No	-1.190	0.069	-1.324, -1.055	<0.001	-0.09	-0.037	0.002	-0.040, -0.034	<0.001	-0.66

β – standardised beta coefficient in the regression models, SE – standard error, 95% CI – 95% confidence interval

In the supplementary file (Table S3 and Table S5 in the **Online Supplementary Document**), we presented the means and standard deviations of the VAS and utility index scores, respectively, by gender, age, region and residency. The respondents living in the eastern developed region had the highest VAS and utility index scores in all age groups. In contrast, those living in the western under-developed region had the lowest VAS and utility index scores. The urban-rural disparities appeared to vary by regions. Urban residents residing in the central region had a higher utility index score for all age groups than their rural counterparts. But in the eastern and western regions, urban residents aged between 15 and 54 years had a lower utility index than their rural counterparts (Table S5 in the **Online Supplementary Document**). Similar results can be found in the medians and IQRs of the VAS and utility index scores presented in the supplementary file (Table S4 and Table S6 in the **Online Supplementary Document**).

## DISCUSSION

To the best of our knowledge, this is the first paper to provide Chinese EQ-5D-3L population norms based on a large sample using the nationally representative preference-based value sets. The population norms, presented in the percentage of reported problems, means (standard deviations), and medians (interquartile ranges) of VAS and utility index scores, can serve as reference for comparative purposes in HRQoL studies and health economic evaluation studies in China.

Overall, the Chinese people have relatively higher utility index scores compared with those from other countries [4,7,11,16,18,19,24,27], although the mean value (0.985) is a bit closer to those in Singapore (0.950) [25] and Korea (0.958) [54]. The European value sets would bring the mean utility index score down to 0.951, which is still high compared with other populations [27,55]. Previous studies using the value sets derived from a small sample of urban populations in big cities may have also underestimated the HRQoL of the Chinese populations (Table S7 in the **Online Supplementary Document**).

Ceiling effects are profound in the Chinese populations (especially in the young groups), with 84.2% reporting no health problems compared with 41.3% in Portugal [19], 47.1% in Poland, 62.4% in Spain [47], 68.0% in Japan [4], and 79.0% in Singapore [25]. Similar findings were also reported in previous studies in China [31,39,44]. Overall, ceiling effects of the EQ-5D-3L instrument were relatively high in Asian countries [4,25,27,31,39,44]. However, the percentage of reported problems in China is not always the lowest in comparison with other countries. Some American/Western European countries (such as Denmark, Germany, Sweden and Switzerland) reported even lower levels of problems in some dimensions (Table S7 in the **Online Supplementary Document**). The differences can be attributable to multiple factors in relation to values, culture, and traditions (eg, tolerance to various health problems) [56,57]. As a result, the use and interpretation of the EQ-5D-3L utility index needs to be cautious. Empirical evidence shows that Asian populations are less likely to report health problems than their European counterparts, inflating the utility index [27]. It is important to note that the percentage of respondents in this study reporting problems on the five dimensions of the EQ-5D-3L are consistently lower compared with the populations in other countries [3,4,7,11,13,16-19,22,24,25,27,47,54,58,59]. Similar to studies undertaken in other countries, pain/discomfort was the most frequently reported problem [11,60]. But only about 12% of the Chinese respondents reported problems in pain/discomfort, much less than those from other countries, which could be as high as 65.0% [3,4,7,11,13,16-19,22,24,25,27,47,54,58,59]. The next frequently reported problems in the Chinese populations were mobility (5.9%) and anxiety/depression (5.3%), again at a level lower than other countries [3,4,7,11,13,16,18,19,22,24,27,47,54,58,59]. The only exceptions are the lower level of reported problems in mobility (3.6%) in the Singapore population [25] and the lower level of reported problems in anxiety/depression in the Netherlands (3.5%) and German (4.3%) populations [11,27].

Unlike the utility index, the average level (80.91) of VAS scores in this study population is similar to those in New Zealand (80.8) [59], Sri Lanka (81.0) [7], Switzerland (81.7) [18] and a previous study in China (80.4) [27], lower than those in Denmark (83.7) [22], Sweden (83.3) [58], the UK (82.8) [13] and the Netherlands (82.0) [17], but higher than those in some other countries (ranging from 71.1 to 80.0) [3,11,19,24]. Further analyses demonstrated a wide distribution of VAS scores in those who reported no health problems, indicating a low discriminatory power of the utility index (Table S7 in the **Online Supplementary Document**).

There exists a gender gap in HRQoL of the Chinese populations. Overall, male respondents were less likely to report health problems than female respondents, resulting in higher VAS and utility index scores. However, after controlling for variations in other factors, the effect size in the differences in gender on the utility index was only 0.05. This phenomenon was also observed in studies in Singapore [25] and other studies in China[31,35,39,61]. Szende and colleagues believe that gender plays a small role in explaining HRQoL [11]. It is worth noting that gender variations in utility index and VAS scores were not always consistent. Women had lower scores in VAS than men, but not in utility index. These inconsistencies may be due to the conceptual differences in the two measurements [62]. VAS reflects a direct individual real-time rating considering all aspects of health; whereas, utility index is an indirect measurement, using past time value sets to estimate current states considering limited dimensions of health [38]. Women may have a relatively higher expectation on health, resulting in lower ratings on VAS [31,44]. Similar to other studies [4,6,7,11,13,16,19,22,24,31,39,60,63], we found that older age is associated with lower HRQoL (with an effect size of 0.91-4.20 on the utility index). China is currently experiencing unprecedented rapid transition to an ageing society as a result of the decades long family planning policy [64], which could lead to an overall decline in HRQoL of the entire population.

Regional and residential disparities in HRQoL in the Chinese populations are evident. Those who resided in the eastern developed region had higher HRQoL than their central and western counterparts, with a medium effect size (0.34-0.55) on the utility index. Rural respondents in this study had higher VAS ratings than their urban counterparts although they were more likely to report problems in mobility, self-care, and usual activities. Unlike the utility index measurement applying past time value sets, VAS scores reflect real time individual subjective ratings. Rural residents may have relatively lower expectation on health, resulting in higher ratings on VAS [31,44]. According to the Guide Book of EQ-5D-3L, both utility index and VAS scores should be presented in result reporting. Young rural residents (≤55 years) had higher utility index scores than their urban counterparts, especially in the eastern and western regions. By contrast, old rural residents (≥65 years) had lower utility index scores than their urban counterparts. These results are consistent with findings reported in previous studies in China and elsewhere [31,35,39,44,61,63]. However, the urban-rural differences revealed in this study is small based on effect size (0.03-0.05). Nevertheless, it is important to note that population mobility is high in China: young people in rural and undeveloped regions are increasingly moving to urban and more developed regions [64,65]. This could exacerbate the aging process in rural areas, lowering the utility index of rural residents.

Socioeconomic gradients in HRQoL as measured by the three indicators (percentage of reported problems, utility index and VAS scores) deserve increased attention. We found that those with a higher socio-economic status (eg, richer, better educated, employed and married) have significantly higher HRQoL than others, after controlling for variations in demographic and health characteristics. These results are consistent with findings from previous studies in China and elsewhere [4,6,7,11,13,16,22,25,31,39,60,63]. The effect size on the utility index reached 0.35-0.76 for education, 0.37-0.74 for income, 0.29-1.11 for employment, and 0.02-0.75 for marital status.

This study adds additional evidence to support the proposed association between physical activity and HRQoL [4,6,7,11,13,16,22,31,39,60,63]. The effect size of regular exercise (0.66) on the utility index shows clinical significance. However, the associations between HRQoL and smoking and drinking are small, albeit statistically significant, failing to reach a clinical meaningful level according to the effect size (0.16 for smoking and 0.13 for drinking). Positive associations between HRQoL and smoking and drinking were reported in previous studies conducted elsewhere in China [66-68].

Limitations: There are several limitations in this study. First, this is a cross-sectional study and no causal relationships should be assumed for the findings. Second, there is a high ceiling effect for the EQ-5D-3L utility index. Although the EQ-5D-5L descriptive system may improve the discriminatory power, its ceiling effect is likely to stay high in the Chinese populations [69,70]. Further studies should examine the cultural responsiveness of the EQ-5D-3L instrument [56] and the appropriateness of the translated wording and phrasing (language) of its descriptive system [71] in the Chinese context. We reported mean values and standard deviations of the EQ-5D utility index in line with other studies despite its high ceiling effect [4,15,24,27,47]. Non-normal distributions of EQ-5D utility index scores are common in all EQ-5D studies: full health status was reported in 79% Singaporean populations, 68% in Japanese populations, and more than 50% of populations in Poland, France and Spain [4,18,24,27,47,60]. Third, the EQ-5D instruments measure limited dimensions of health due to a small number (five) of items. The small item number made it easier to generate a utility index score, which is often absent from a more comprehensive instrument (for example the SF-36) [38,72,73]. However, the implications of the utility index need to be interpreted with caution, and in conjunction with analyses of the problems reported and VAS scores.

## CONCLUSIONS

This paper provides population norms for the EQ-5D-3L in China stratified by age, gender and region based on the 2018 population preference-based value sets derived from a national representative sample. The norms can be used as a reference for health economic evaluation studies. Overall, 15.8% of respondents reported a health problem, with pain/discomfort being the most commonly reported problem. Compared with other populations, the Chinese people have high scores in VAS and utility index. Those who are richer, better educated, employed, married, and live in developed areas have higher scores in both VAS and utility index than others. Further studies are needed to explore the underlying reasons of the sociodemographic differences.

Given the high ceiling effect and low discriminatory power of the utility index, cautions should be taken in presenting and interpreting mean values of the utility index. The meaning and implications of the EQ-5D-3L utility index need to be interpreted in conjunction with other indicators, including the nature and number of problems reported, the distributional position of the health state, and the VAS scores. However, we acknowledge that the ceiling effect of the utility index is less serious in some subpopulations, such as the elderly and those with existing illness conditions, as revealed in this study. This suggests that it may be more appropriate to use the EQ-5D-3L instrument in those subpopulations rather than the entire general population. We advocate use of the percentile indicators for presenting population-based results of the EQ-5D-3L utility index and further studies into the health state descriptive system tailored to the specific context of the Chinese populations.

## Additional material

Online Supplementary Document
