# Supplementary material for: Population norms for the EQ-5D-3L in China derived from the 2013 National Health Services Survey
Source: J Glob Health. 2021 Feb 11;11:08001. doi: 10.7189/jogh.11.08001 (PMC7916444; doi:10.7189/jogh.11.08001)
Supplement: Online Supplementary Document [file jogh-11-08001-s001.pdf]

Figures

Figure S1 Correlation between EQ-5D-3L VAS scores and utility index in male respondents (r=0.4577, $P<0.05$).

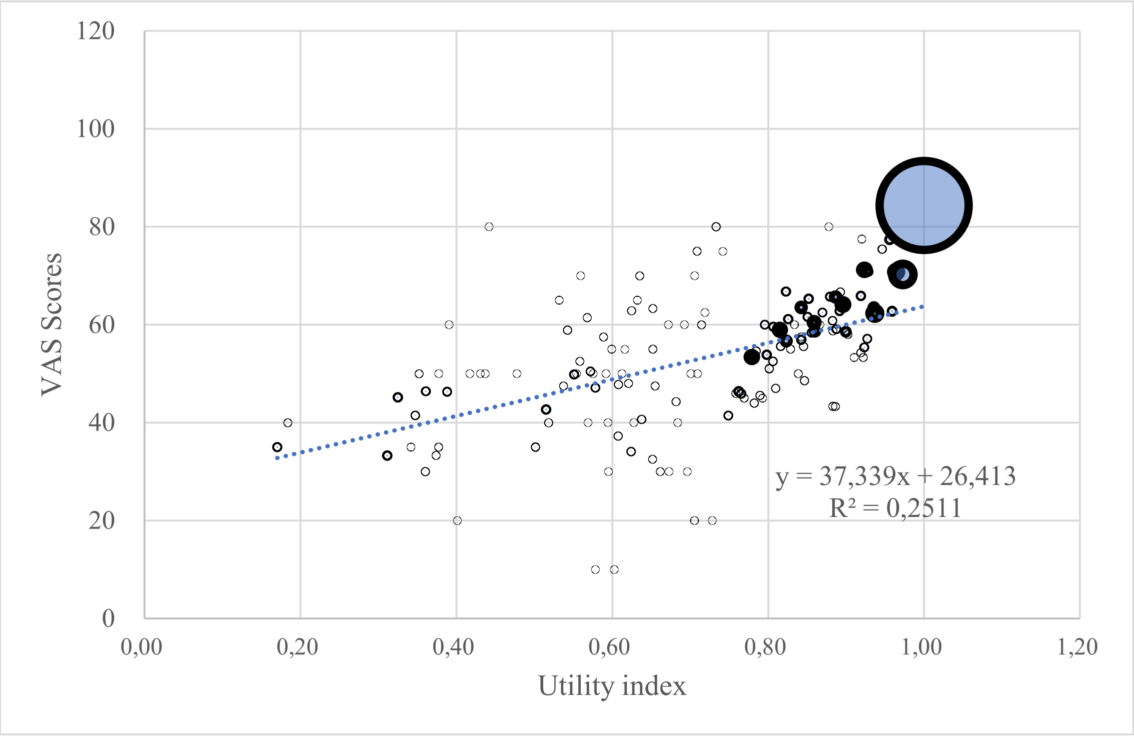

Figure S2 Correlation between EQ-5D-3L VAS scores and utility index in female respondents (r=0.4501, P<0.05).

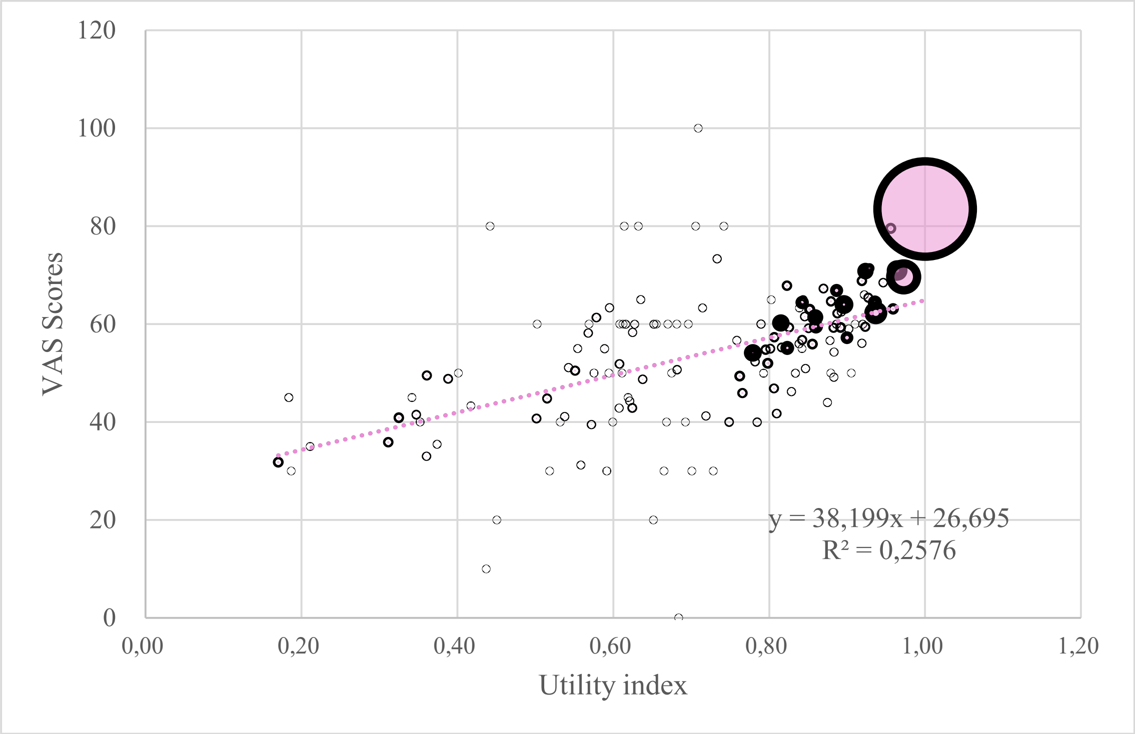

Figure S3 Correlation between EQ-5D-3L VAS scores and utility index in 15-24 years age (r=0.2403, P<0.05).

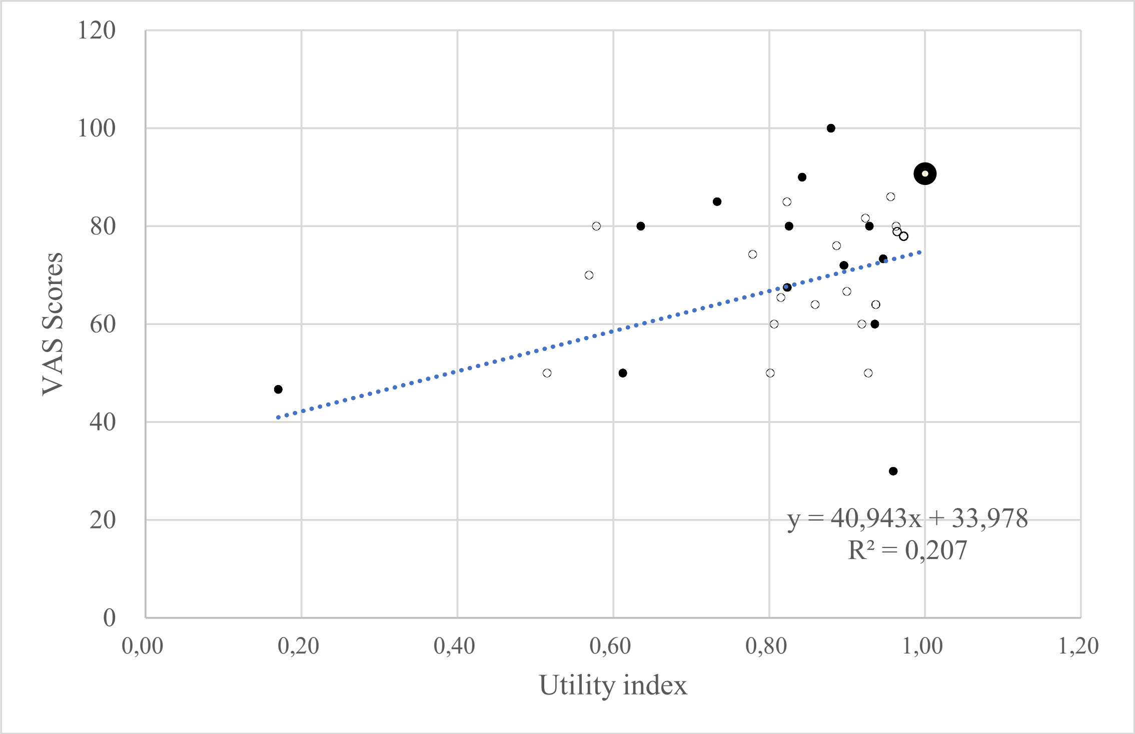

Figure S4 Correlation between EQ-5D-3L VAS scores and utility index in 25-34 years age (r=0.3070, P<0.05).

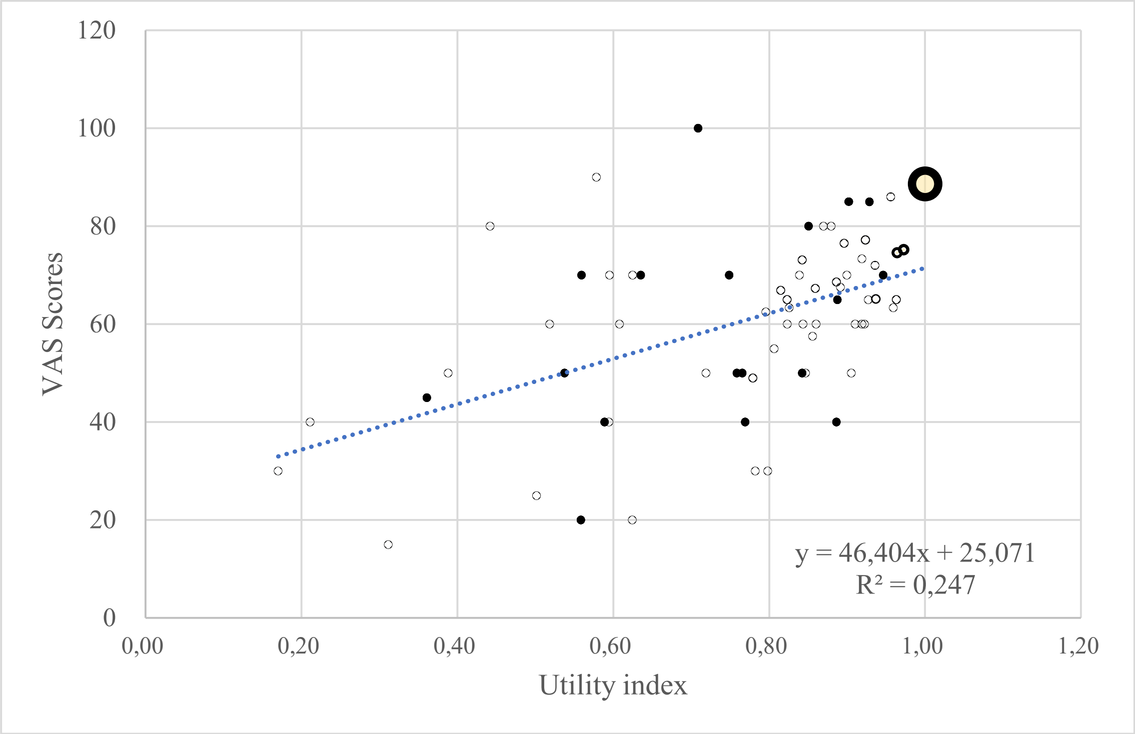

Figure S5 Correlation between EQ-5D-3L VAS scores and utility index in 35-44 years age (r=0.3620, P<0.05).

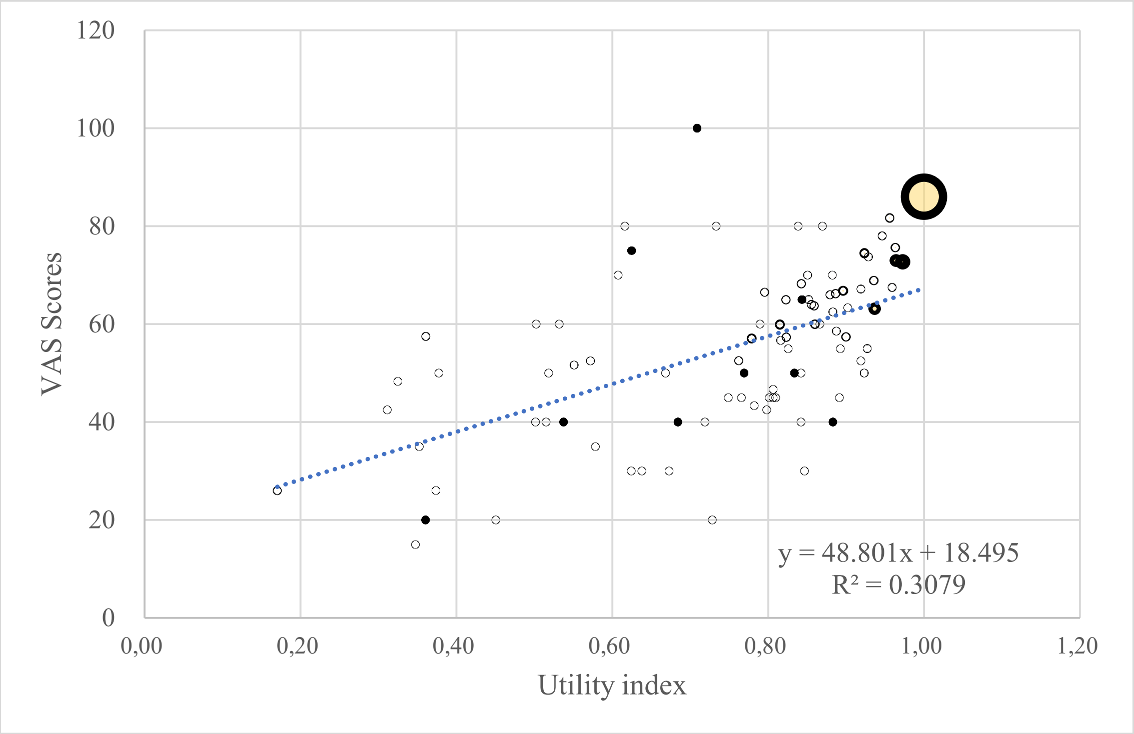

Figure S6 Correlation between EQ-5D-3L VAS scores and utility index in 45-54 years age (r=0.3620, P<0.05).

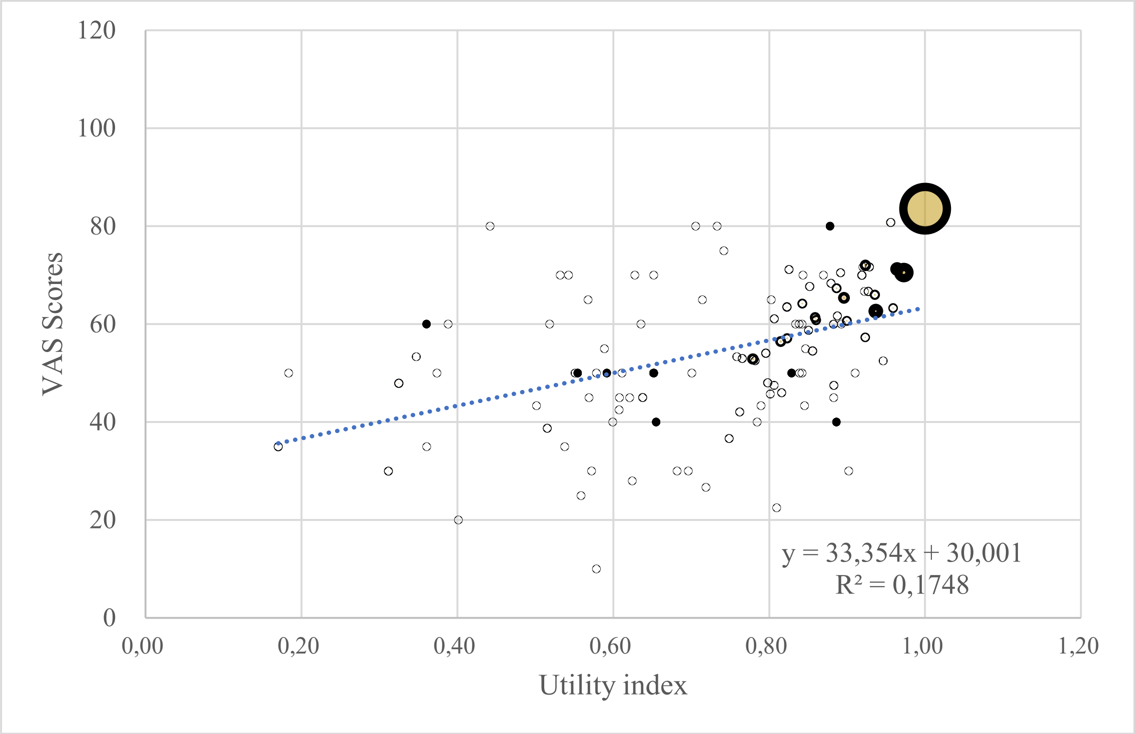

Figure S7 Correlation between EQ-5D-3L VAS scores and utility index in 55-64 years age (r=0.4225, P<0.05).

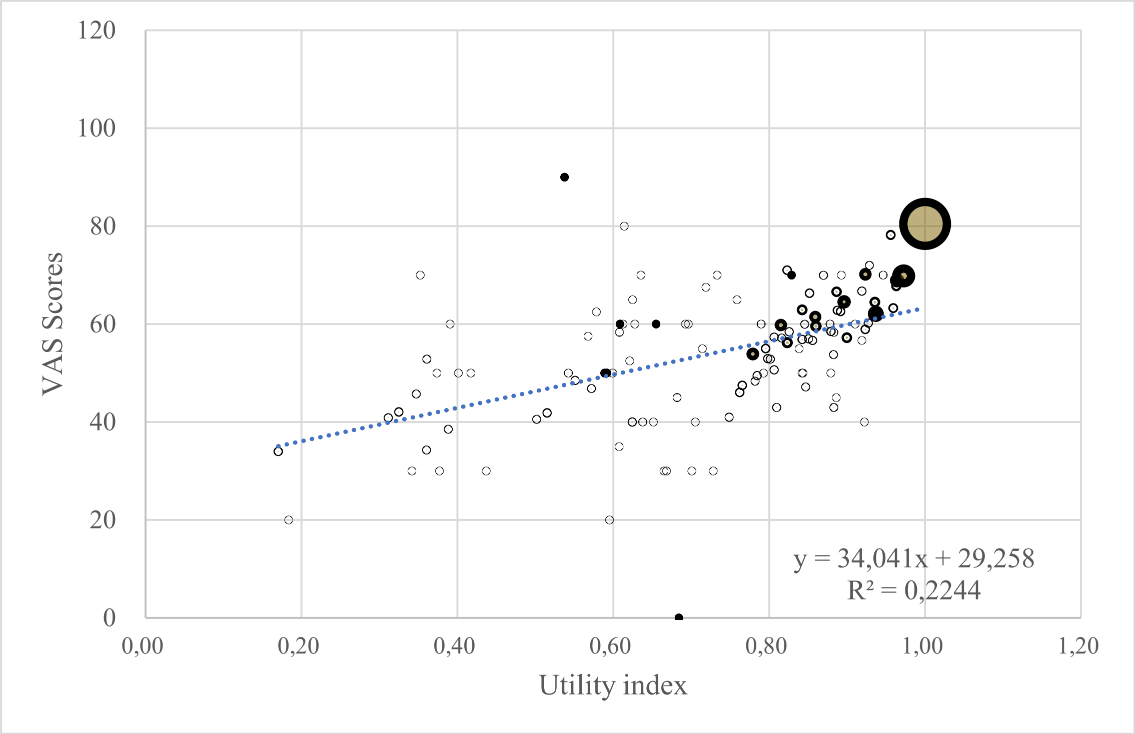

Figure S8 Correlation between EQ-5D-3L VAS scores and utility index in 65-74 years age (r=0.4564, P<0.05).

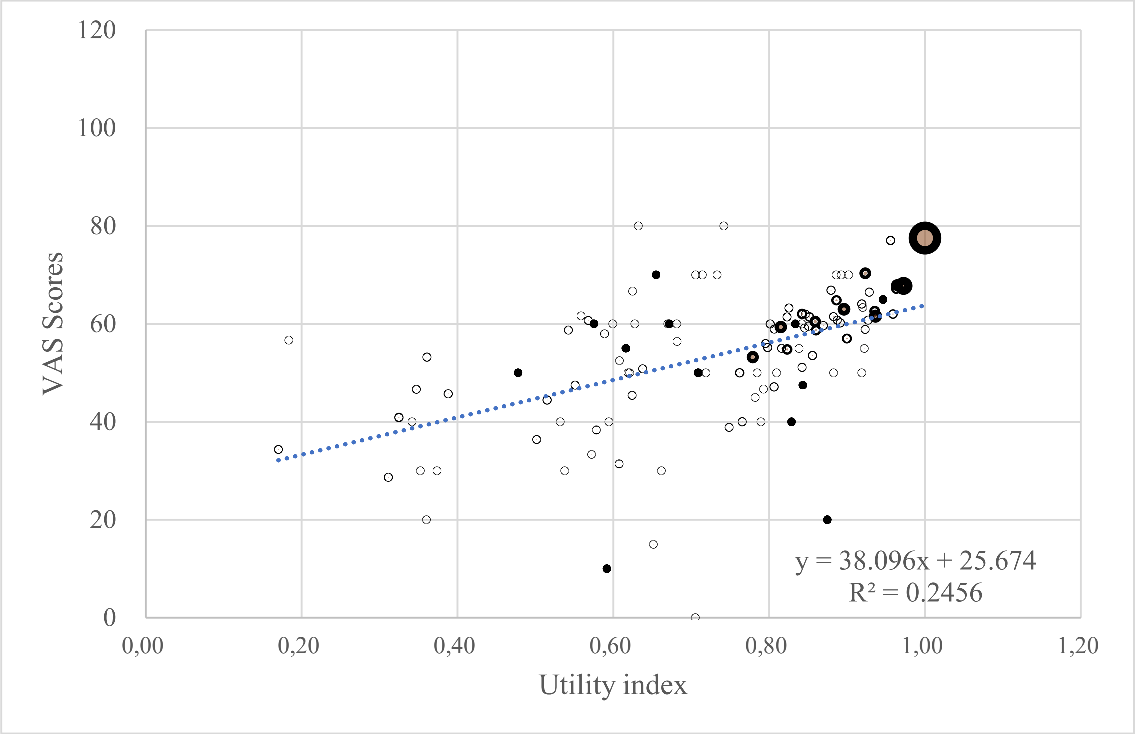

Figure S9 Correlation between EQ-5D-3L VAS scores and utility index in 75 and above years age (r=0.4564, P<0.05).

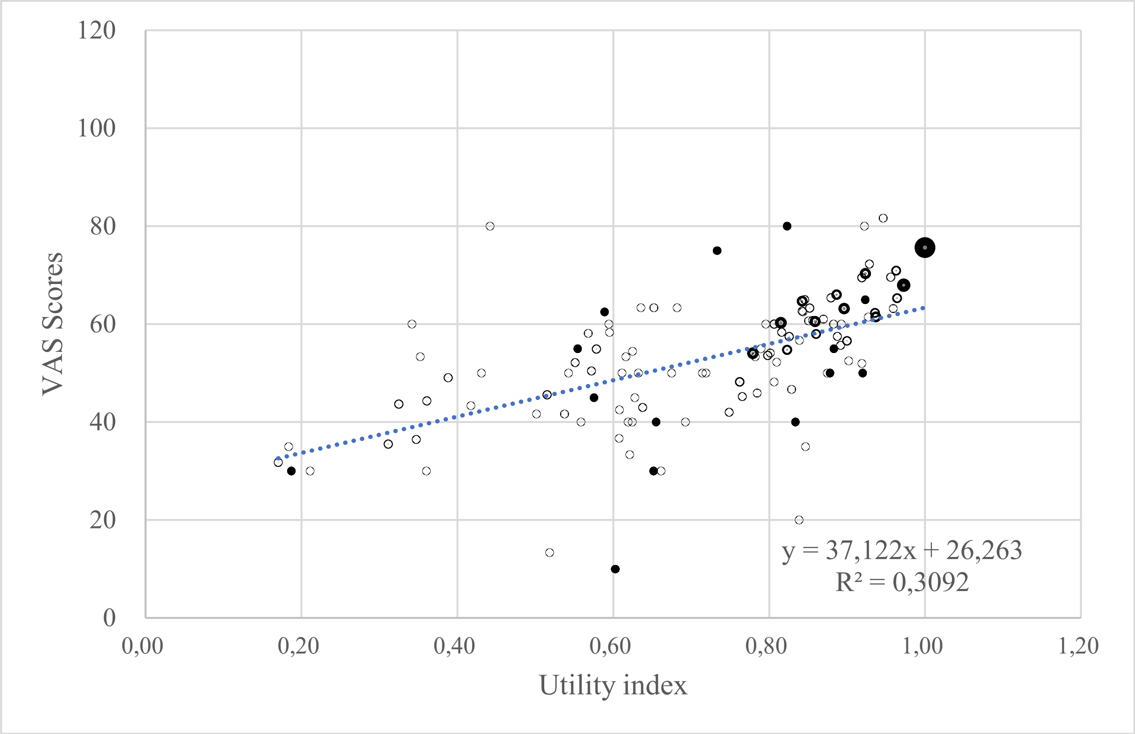

Tables

Table S1 Distribution of EQ-5D-3L health states in Chinese population (n=188720)

	Utility index score	Frequency	Percentage (%)	Cumulative percentage (%)
33333	0.17	93	0.05	0.05
33332	0.31	79	0.04	0.09
33331	0.35	40	0.02	0.11
33323	0.18	10	0.01	0.12
33322	0.33	116	0.06	0.18
33321	0.36	59	0.03	0.21
33313	0.21	2	0.00	0.21
33312	0.35	7	0.00	0.22
33311	0.39	52	0.03	0.24
33233	0.19	1	0.00	0.24
33222	0.34	4	0.00	0.25
33221	0.38	1	0.00	0.25
33111	0.44	3	0.00	0.25
32333	0.42	4	0.00	0.25
32332	0.56	8	0.00	0.25
32331	0.59	3	0.00	0.26
32323	0.43	2	0.00	0.26
32322	0.57	29	0.02	0.27
32321	0.61	25	0.01	0.29
32312	0.60	3	0.00	0.29
32311	0.64	7	0.00	0.29
32232	0.58	3	0.00	0.29
32231	0.61	2	0.00	0.29
32222	0.59	11	0.01	0.30
32221	0.63	16	0.01	0.31
32211	0.65	7	0.00	0.31
32132	0.61	2	0.00	0.31
32121	0.66	1	0.00	0.31
31332	0.60	1	0.00	0.31
31322	0.62	5	0.00	0.32
31321	0.65	2	0.00	0.32
31313	0.50	1	0.00	0.32
31211	0.70	2	0.00	0.32
31122	0.67	1	0.00	0.32
31121	0.71	2	0.00	0.32
31111	0.73	15	0.01	0.33
23333	0.36	19	0.01	0.34
23332	0.50	33	0.02	0.36
23331	0.54	13	0.01	0.36
23323	0.37	7	0.00	0.37
23322	0.52	72	0.04	0.40
23321	0.55	61	0.03	0.44
23313	0.40	2	0.00	0.44
23312	0.54	18	0.01	0.45
23311	0.58	38	0.02	0.47
23233	0.38	2	0.00	0.47
23232	0.52	7	0.00	0.47
23231	0.55	3	0.00	0.47
23223	0.39	1	0.00	0.47
23222	0.53	3	0.00	0.48
23221	0.57	18	0.01	0.49
23212	0.56	1	0.00	0.49
23211	0.60	8	0.00	0.49

23122	0.57	3	0.00	0.49
23111	0.63	3	0.00	0.49
22333	0.61	18	0.01	0.50
22332	0.75	42	0.02	0.53
22331	0.79	25	0.01	0.54
22323	0.62	12	0.01	0.54
22322	0.76	127	0.07	0.61
22321	0.80	109	0.06	0.67
22312	0.79	15	0.01	0.68
22311	0.83	75	0.04	0.72
22233	0.62	41	0.02	0.74
22232	0.77	75	0.04	0.78
22231	0.80	39	0.02	0.80
22223	0.64	27	0.01	0.81
22222	0.78	1431	0.76	1.57
22221	0.82	1372	0.73	2.30
22212	0.81	74	0.04	2.34
22211	0.84	518	0.27	2.61
22133	0.66	1	0.00	2.61
22132	0.80	1	0.00	2.61
22131	0.84	7	0.00	2.62
22123	0.68	1	0.00	2.62
22122	0.82	39	0.02	2.64
22121	0.85	118	0.06	2.70
22112	0.84	13	0.01	2.71
22111	0.88	85	0.05	2.75
21333	0.65	5	0.00	2.76
21332	0.79	5	0.00	2.76
21331	0.83	10	0.01	2.76
21323	0.67	2	0.00	2.76
21322	0.81	48	0.03	2.79
21321	0.84	68	0.04	2.83
21313	0.69	2	0.00	2.83
21312	0.83	5	0.00	2.83
21311	0.87	46	0.02	2.85
21233	0.67	2	0.00	2.86
21232	0.81	27	0.01	2.87
21231	0.85	25	0.01	2.88
21223	0.68	17	0.01	2.89
21222	0.82	497	0.26	3.16
21221	0.86	961	0.51	3.66
21213	0.71	1	0.00	3.67
21212	0.85	49	0.03	3.69
21211	0.89	456	0.24	3.93
21133	0.71	2	0.00	3.93
21132	0.85	18	0.01	3.94
21131	0.88	38	0.02	3.96
21123	0.72	10	0.01	3.97
21122	0.86	492	0.26	4.23
21121	0.90	1716	0.91	5.14
21112	0.89	63	0.03	5.17
21111	0.92	1292	0.68	5.86
13333	0.44	2	0.00	5.86
13332	0.58	1	0.00	5.86
13331	0.61	1	0.00	5.86
13323	0.45	1	0.00	5.86
13322	0.59	3	0.00	5.86

13321	0.63	5	0.00	5.86
13313	0.48	1	0.00	5.86
13312	0.62	2	0.00	5.87
13311	0.66	5	0.00	5.87
13222	0.61	1	0.00	5.87
13211	0.67	1	0.00	5.87
13121	0.68	1	0.00	5.87
13112	0.67	1	0.00	5.87
13111	0.71	3	0.00	5.87
12333	0.68	2	0.00	5.87
12322	0.84	5	0.00	5.88
12321	0.88	5	0.00	5.88
12312	0.87	1	0.00	5.88
12311	0.90	10	0.01	5.88
12233	0.70	2	0.00	5.88
12232	0.84	6	0.00	5.89
12231	0.88	4	0.00	5.89
12223	0.71	7	0.00	5.89
12222	0.86	89	0.05	5.94
12221	0.89	91	0.05	5.99
12213	0.74	2	0.00	5.99
12212	0.88	11	0.01	6.00
12211	0.92	94	0.05	6.05
12132	0.88	1	0.00	6.05
12122	0.89	14	0.01	6.05
12121	0.93	62	0.03	6.09
12112	0.92	7	0.00	6.09
12111	0.96	157	0.08	6.17
11333	0.73	2	0.00	6.17
11331	0.91	1	0.00	6.18
11322	0.88	12	0.01	6.18
11321	0.92	16	0.01	6.19
11313	0.77	2	0.00	6.19
11312	0.91	4	0.00	6.19
11311	0.95	24	0.01	6.21
11232	0.89	6	0.00	6.21
11231	0.92	8	0.00	6.21
11223	0.76	8	0.00	6.22
11222	0.90	302	0.16	6.38
11221	0.94	474	0.25	6.63
11212	0.93	57	0.03	6.66
11211	0.96	359	0.19	6.85
11133	0.78	23	0.01	6.86
11132	0.92	63	0.03	6.89
11131	0.96	156	0.08	6.98
11123	0.80	65	0.03	7.01
11122	0.94	3102	1.64	8.66
11121	0.97	11069	5.87	14.52
11113	0.82	89	0.05	14.57
11112	0.96	2347	1.24	15.81
11111	1.00	158880	84.19	100.00

Table S2 EQ-5D-3L utility index and VAS scores (Median and Interquartile Range IQR) by socio-demographic characteristics of respondents

Characteristics of respondents		n	%	VAS score				Utility index			
				Median	IQR (Q1, Q3)*	Z/χ2	P	Median	IQR (Q1, Q3)	Z/χ2	P
**Demographic**											
Gender											
	Male	89830	47.60	80.00	(80.00, 90.00)	*20.58*	*<0.001*	1.000	(1.000, 1.000)	*16.73*	*<0.001*
	Female	98890	52.40	80.00	(70.00, 90.00)			1.000	(1.000, 1.000)		
Age											
	15-24	14094	7.47	90.00	(90.00, 96.00)			1.000	(1.000, 1.000)		
	25-34	24347	12.90	90.00	(80.00, 90.00)			1.000	(1.000, 1.000)		
	35-44	35081	18.59	90.00	(80.00, 90.00)			1.000	(1.000, 1.000)		
	45-54	41000	21.73	80.00	(80.00, 90.00)	*39020.08*	*<0.001*	1.000	(1.000, 1.000)	*20166.43*	*<0.001*
	55-64	40532	21.48	80.00	(70.00, 90.00)			1.000	(1.000, 1.000)		
	65-74	22138	11.73	75.00	(60.00, 80.00)			1.000	(0.973, 1.000)		
	75+	11528	6.11	70.00	(60.00, 80.00)			1.000	(0.896, 1.000)		
**Residency**											
Location											
	Urban	94064	49.84	80.00	(70.00, 90.00)	*-12.18*	*<0.001*	1.000	(1.000, 1.000)	*0.29*	*0.774*
	Rural	94656	50.16	80.00	(75.00, 90.00)			1.000	(1.000, 1.000)		
Region											
	Eastern	66575	35.28	85.00	(80.00, 90.00)			1.000	(1.000, 1.000)		
	Central	58306	30.90	80.00	(70.00, 90.00)	*870.89*	*<0.001*	1.000	(1.000, 1.000)	*218.20*	*<0.001*
	Western	63839	33.83	80.00	(70.00, 90.00)			1.000	(1.000, 1.000)		
**Socio-economic**											
Educational attainment											
	Illiterate	22709	12.03	80.00	(60.00, 80.00)			1.000	(0.973, 1.000)		
	Primary school	48953	25.94	80.00	(70.00, 90.00)			1.000	(1.000, 1.000)		
	Junior middle school	65877	34.91	89.00	(80.00, 90.00)	*14357.72*	*<0.001*	1.000	(1.000, 1.000)	*9053.04*	*<0.001*
	Senior middle school	32435	17.19	90.00	(80.00, 90.00)			1.000	(1.000, 1.000)		
	University/college or above	18746	9.93	90.00	(80.00, 90.00)			1.000	(1.000, 1.000)		

Local ranking of average household income											
	Lowest (<percentile 20)	35702	18.93	80.00	(70.00, 90.00)			1.000	(1.000, 1.000)		
	Low (percentile 20-39)	35471	18.80	80.00	(70.00, 90.00)			1.000	(1.000, 1.000)		
	Middle (percentile 40-59)	37124	19.68	80.00	(80.00, 90.00)	*2313.12*	*<0.001*	1.000	(1.000, 1.000)	*2113.31*	*<0.001*
	High (percentile 60-79)	39084	20.72	85.00	(80.00, 90.00)			1.000	(1.000, 1.000)		
	Highest (>=percentile 80)	41251	21.87	85.00	(80.00, 90.00)			1.000	(1.000, 1.000)		
Employment											
	Employed	127614	67.62	85.00	(80.00, 90.00)			1.000	(1.000, 1.000)		
	Retired	27274	14.45	80.00	(70.00, 85.00)	*15565.41*	*<0.001*	1.000	(1.000, 1.000)	*9099.38*	*<0.001*
	Student	4747	2.52	90.00	(90.00, 98.00)			1.000	(1.000, 1.000)		
	Unemployed	29085	15.41	80.00	(65.00, 90.00)			1.000	(0.937, 1.000)		
Marital status											
	Never married/Single	17131	9.08	90.00	(85.00, 95.00)			1.000	(1.000, 1.000)		
	Married	155755	82.53	80.00	(75.00, 90.00)	*12270.47*	*<0.001*	1.000	(1.000, 1.000)	*6859.82*	*<0.001*
	Widowed	12932	6.85	70.00	(60.00, 80.00)			1.000	(0.937, 1.000)		
	Divorced	2898	1.54	80.00	(70.00, 90.00)			1.000	(1.000, 1.000)		
**Illness condition**											
Two-week morbidity											
	Yes	44986	23.84	75.00	(60.00, 80.00)	*-139.95*	*<0.001*	1.000	(0.973, 1.000)	*-127.24*	*<0.001*
	No	143734	76.16	90.00	(80.00, 90.00)			1.000	(1.000, 1.000)		
Chronic disease											
	Yes	50698	26.86	75.00	(60.00, 80.00)	*-160.43*	*<0.001*	1.000	(0.973, 1.000)	*-138.90*	*<0.001*
	No	138022	73.14	90.00	(80.00, 90.00)			1.000	(1.000, 1.000)		
One-year hospital admission											
	Yes	17016	9.02	80.00	(60.00, 90.00)	*-68.07*	*<0.001*	1.000	(0.973, 1.000)	*-74.53*	*<0.001*
	No	171681	90.98	80.00	(80.00, 90.00)			1.000	(1.000, 1.000)		
**Lifestyle and behaviors**											
Smoking											
	Yes	49208	26.09	80.00	(80.00, 90.00)	*10.18*	*<0.001*	1.000	(1.000, 1.000)	*14.92*	*<0.001*
	No	139399	73.91	80.00	(70.00, 90.00)			1.000	(1.000, 1.000)		
Drinking											

	Yes	44005	23.32	85.00	(80.00, 90.00)	*19.55*	*<0.001*	1.000	(1.000, 1.000)	*19.63*	*<0.001*
	No	144706	76.68	80.00	(70.00, 90.00)			1.000	(1.000, 1.000)		
Physical exercise											
	Yes	55843	29.67	80.00	(70.00, 90.00)	*-5.67*	*<0.001*	1.000	(1.000, 1.000)	*8.98*	*<0.001*
	No	132372	70.33	80.00	(70.00, 90.00)			1.000	(1.000, 1.000)		
**Total**		188720	100.00	80.00	(70.00, 90.00)	—	—	1.000	(1.000, 1.000)	—	—

*Note: Q1 indicates the 25th percentiles and Q3 indicates the 75th percentiles. Wilcoxon (Mann-Whitney) rank-sum test for two sample (Z), and Kruskal-Wallis equality-of-populations rank test for three and above samples (χ^2).

Table S3 VAS scores (Mean, SD) of respondents by gender and age

		Urban								Rural								Total							
		Eastern		Central		Western		Total		Eastern		Central		Western		Total		Eastern		Central		Western		Total	
		Mean	SD	Mean	SD	Mean	SD	Mean	SD	Mean	SD	Mean	SD	Mean	SD	Mean	SD	Mean	SD	Mean	SD	Mean	SD	Mean	SD
All	Total	81.94	13.28	80.58	13.86	79.11	14.08	80.59	13.78	82.31	13.22	80.59	14.12	80.71	13.76	81.23	13.71	82.12	13.25	80.59	13.99	79.94	13.94	80.91	13.74
	15-24	91.48	7.96	91.13	8.10	88.58	9.25	90.36	8.58	91.60	7.66	90.75	8.38	89.48	8.21	90.43	8.14	91.53	7.82	90.94	8.24	89.10	8.67	90.40	8.35
	25-34	88.42	9.11	88.23	9.15	86.31	9.68	87.70	9.35	89.43	8.81	88.51	9.91	87.38	9.49	88.37	9.42	88.85	9.00	88.35	9.49	86.86	9.60	88.01	9.39
	35-44	85.59	10.89	84.60	11.50	82.48	12.16	84.14	11.63	86.35	10.44	85.16	11.36	84.29	11.12	85.20	11.03	85.98	10.67	84.88	11.43	83.43	11.66	84.68	11.34
	45-54	82.05	12.48	80.66	13.55	79.16	13.18	80.61	13.13	83.50	11.84	81.40	13.00	80.85	12.61	81.97	12.52	82.86	12.14	81.05	13.27	80.06	12.90	81.35	12.82
	55-64	78.89	12.89	76.83	13.48	75.42	13.91	77.21	13.46	79.21	12.99	76.65	13.84	76.00	13.95	77.37	13.65	79.05	12.94	76.74	13.67	75.72	13.93	77.29	13.56
	65-74	75.82	14.12	73.14	13.98	71.10	14.62	73.49	14.36	74.61	13.74	72.10	14.62	70.99	14.90	72.63	14.49	75.26	13.96	72.66	14.29	71.05	14.76	73.08	14.43
	75+	71.39	15.15	69.91	14.76	67.86	15.49	69.88	15.21	69.14	15.21	67.77	15.52	66.38	15.40	67.95	15.39	70.40	15.22	68.98	15.13	67.24	15.47	69.05	15.32
Males	Total	82.56	13.06	80.94	13.90	79.92	13.90	81.20	13.64	82.95	12.93	81.06	13.90	81.61	13.38	81.90	13.41	82.75	13.00	81.00	13.90	80.82	13.65	81.56	13.53
	15-24	91.95	7.80	91.54	8.41	89.25	8.91	90.90	8.45	92.06	7.47	91.28	7.92	89.74	8.25	90.78	8.02	92.00	7.65	91.41	8.17	89.55	8.52	90.84	8.22
	25-34	88.83	8.82	88.53	9.28	86.92	9.49	88.16	9.20	89.80	8.88	88.68	10.14	88.02	9.33	88.78	9.43	89.24	8.86	88.60	9.67	87.52	9.42	88.45	9.31
	35-44	85.81	10.78	85.00	11.48	83.24	12.00	84.62	11.50	86.86	10.25	85.62	11.34	85.03	10.76	85.77	10.82	86.33	10.53	85.31	11.41	84.18	11.40	85.20	11.18
	45-54	82.54	12.62	80.93	13.65	80.34	12.93	81.28	13.10	84.24	11.75	82.19	12.67	81.98	12.27	82.85	12.26	83.50	12.17	81.60	13.15	81.25	12.59	82.15	12.66
	55-64	79.63	12.78	77.35	13.82	76.67	13.39	78.04	13.35	80.24	12.62	77.43	13.62	77.66	13.21	78.52	13.20	79.94	12.70	77.40	13.72	77.20	13.30	78.29	13.27
	65-74	76.41	13.96	74.10	13.74	72.03	14.66	74.29	14.23	75.57	13.42	73.22	14.26	72.12	14.76	73.69	14.21	76.00	13.71	73.67	14.00	72.07	14.71	73.99	14.22
	75+	72.95	14.86	71.18	14.86	68.93	15.87	71.15	15.26	70.69	14.78	69.43	15.43	67.59	15.79	69.40	15.33	71.92	14.86	70.42	15.13	68.37	15.84	70.38	15.31
Females	Total	81.39	13.44	80.28	13.82	78.41	14.21	80.07	13.87	81.73	13.45	80.16	14.30	79.82	14.06	80.59	13.95	81.55	13.44	80.22	14.05	79.12	14.15	80.32	13.91
	15-24	91.04	8.09	90.79	7.82	87.99	9.50	89.89	8.66	91.17	7.81	90.29	8.74	89.21	8.16	90.09	8.25	91.10	7.96	90.55	8.29	88.67	8.79	89.99	8.45
	25-34	88.07	9.34	88.01	9.05	85.85	9.81	87.34	9.46	89.12	8.74	88.36	9.72	86.77	9.61	88.02	9.40	88.52	9.10	88.16	9.34	86.30	9.72	87.64	9.44
	35-44	85.39	10.98	84.24	11.51	81.80	12.26	83.71	11.72	85.92	10.58	84.77	11.37	83.62	11.40	84.70	11.18	85.66	10.78	84.51	11.44	82.75	11.85	84.22	11.46
	45-54	81.62	12.33	80.44	13.48	78.18	13.31	80.06	13.13	82.82	11.87	80.70	13.25	79.77	12.83	81.16	12.70	82.28	12.09	80.58	13.36	79.00	13.09	80.64	12.91
	55-64	78.23	12.96	76.38	13.17	74.29	14.27	76.48	13.52	78.19	13.27	75.92	14.01	74.31	14.48	76.24	13.99	78.21	13.11	76.15	13.60	74.30	14.37	76.36	13.75
	65-74	75.25	14.24	72.27	14.14	70.21	14.53	72.73	14.45	73.56	14.02	70.83	14.92	69.71	14.96	71.44	14.71	74.50	14.17	71.64	14.50	69.97	14.73	72.15	14.58
	75+	70.15	15.27	68.59	14.54	66.84	15.07	68.73	15.07	67.75	15.45	66.00	15.42	65.19	14.93	66.53	15.33	69.12	15.39	67.48	14.98	66.17	15.03	67.80	15.22

Table S4 VAS scores (Median and Interquartile Range IQR) of respondents by gender and age

		Eastern		Central		Western		Total	
		Mean	IQR (Q1, Q3)	Mean	IQR (Q1, Q3)	Mean	IQR (Q1, Q3)	Mean	IQR (Q1, Q3)
**Urban & rural**									
All	Total	85.00	(80.00, 90.00)	80.00	(70.00, 90.00)	80.00	(70.00, 90.00)	80.00	(70.00, 90.00)
	15-24	90.00	(90.00, 100.00)	90.00	(90.00, 100.00)	90.00	(90.00, 95.00)	90.00	(90.00, 96.00)
	25-34	90.00	(85.00, 95.00)	90.00	(80.00, 90.00)	90.00	(80.00, 90.00)	90.00	(80.00, 90.00)
	35-44	90.00	(80.00, 90.00)	90.00	(80.00, 90.00)	90.00	(80.00, 90.00)	90.00	(80.00, 90.00)
	45-54	85.00	(80.00, 90.00)	80.00	(80.00, 90.00)	80.00	(70.00, 90.00)	80.00	(80.00, 90.00)
	55-64	80.00	(70.00, 90.00)	80.00	(70.00, 90.00)	80.00	(70.00, 85.00)	80.00	(70.00, 90.00)
	65-74	80.00	(70.00, 85.00)	75.00	(60.00, 80.00)	70.00	(60.00, 80.00)	75.00	(60.00, 80.00)
	75+	70.00	(60.00, 80.00)	70.00	(60.00, 80.00)	70.00	(60.00, 80.00)	70.00	(60.00, 80.00)
Males	Total	85.00	(80.00, 90.00)	80.00	(70.00, 90.00)	80.00	(70.00, 90.00)	80.00	(80.00, 90.00)
	15-24	90.00	(90.00, 100.00)	90.00	(90.00, 100.00)	90.00	(90.00, 95.00)	90.00	(90.00, 99.00)
	25-34	90.00	(85.00, 95.00)	90.00	(85.00, 95.00)	90.00	(80.00, 90.00)	90.00	(85.00, 90.00)
	35-44	90.00	(80.00, 90.00)	90.00	(80.00, 90.00)	90.00	(80.00, 90.00)	90.00	(80.00, 90.00)
	45-54	90.00	(80.00, 90.00)	80.00	(80.00, 90.00)	80.00	(80.00, 90.00)	85.00	(80.00, 90.00)
	55-64	80.00	(70.00, 90.00)	80.00	(70.00, 90.00)	80.00	(70.00, 90.00)	80.00	(70.00, 90.00)
	65-74	80.00	(70.00, 85.00)	80.00	(70.00, 80.00)	70.00	(60.00, 80.00)	80.00	(70.00, 80.00)
	75+	70.00	(60.00, 80.00)	70.00	(60.00, 80.00)	70.00	(60.00, 80.00)	70.00	(60.00, 80.00)
Females	Total	80.00	(75.00, 90.00)	80.00	(70.00, 90.00)	80.00	(70.00, 90.00)	80.00	(70.00, 90.00)
	15-24	90.00	(90.00, 100.00)	90.00	(90.00, 99.00)	90.00	(85.00, 92.00)	90.00	(90.00, 95.00)
	25-34	90.00	(80.00, 95.00)	90.00	(80.00, 90.00)	90.00	(80.00, 90.00)	90.00	(80.00, 90.00)
	35-44	90.00	(80.00, 90.00)	90.00	(80.00, 90.00)	85.00	(80.00, 90.00)	90.00	(80.00, 90.00)
	45-54	80.00	(80.00, 90.00)	80.00	(75.00, 90.00)	80.00	(70.00, 90.00)	80.00	(75.00, 90.00)
	55-64	80.00	(70.00, 90.00)	80.00	(70.00, 85.00)	80.00	(70.00, 85.00)	80.00	(70.00, 90.00)
	65-74	80.00	(70.00, 80.00)	70.00	(60.00, 80.00)	70.00	(60.00, 80.00)	70.00	(60.00, 80.00)
	75+	70.00	(60.00, 80.00)	70.00	(60.00, 80.00)	70.00	(60.00, 80.00)	70.00	(60.00, 80.00)
**Urban**									
All	Total	82.00	(80.00, 90.00)	80.00	(70.00, 90.00)	80.00	(70.00, 90.00)	80.00	(70.00, 90.00)
	15-24	90.00	(90.00, 100.00)	90.00	(90.00, 100.00)	90.00	(85.00, 92.00)	90.00	(90.00, 96.00)
	25-34	90.00	(82.00, 95.00)	90.00	(80.00, 90.00)	90.00	(80.00, 90.00)	90.00	(80.00, 90.00)
	35-44	90.00	(80.00, 90.00)	90.00	(80.00, 90.00)	85.00	(80.00, 90.00)	90.00	(80.00, 90.00)
	45-54	80.00	(80.00, 90.00)	80.00	(70.00, 90.00)	80.00	(70.00, 90.00)	80.00	(75.00, 90.00)
	55-64	80.00	(70.00, 90.00)	80.00	(70.00, 90.00)	80.00	(70.00, 85.00)	80.00	(70.00, 90.00)
	65-74	80.00	(70.00, 85.00)	75.00	(60.00, 80.00)	70.00	(60.00, 80.00)	80.00	(65.00, 80.00)
	75+	70.00	(60.00, 80.00)	70.00	(60.00, 80.00)	70.00	(60.00, 80.00)	70.00	(60.00, 80.00)
Males	Total	85.00	(80.00, 90.00)	80.00	(70.00, 90.00)	80.00	(70.00, 90.00)	80.00	(75.00, 90.00)
	15-24	90.00	(90.00, 100.00)	90.00	(90.00, 100.00)	90.00	(90.00, 95.00)	90.00	(90.00, 99.00)
	25-34	90.00	(85.00, 95.00)	90.00	(85.00, 90.00)	90.00	(80.00, 90.00)	90.00	(80.00, 90.00)
	35-44	90.00	(80.00, 90.00)	90.00	(80.00, 90.00)	90.00	(80.00, 90.00)	90.00	(80.00, 90.00)
	45-54	85.00	(80.00, 90.00)	80.00	(80.00, 90.00)	80.00	(75.00, 90.00)	80.00	(80.00, 90.00)
	55-64	80.00	(70.00, 90.00)	80.00	(70.00, 90.00)	80.00	(70.00, 90.00)	80.00	(70.00, 90.00)
	65-74	80.00	(70.00, 85.00)	80.00	(70.00, 80.00)	70.00	(60.00, 80.00)	80.00	(70.00, 80.00)
	75+	75.00	(60.00, 80.00)	70.00	(60.00, 80.00)	70.00	(60.00, 80.00)	70.00	(60.00, 80.00)
Females	Total	80.00	(75.00, 90.00)	80.00	(70.00, 90.00)	80.00	(70.00, 90.00)	80.00	(70.00, 90.00)

	15-24	90.00	(90.00, 100.00)	90.00	(90.00, 98.00)	90.00	(80.00, 90.00)	90.00	(90.00, 95.00)
	25-34	90.00	(80.00, 92.00)	90.00	(80.00, 90.00)	90.00	(80.00, 90.00)	90.00	(80.00, 90.00)
	35-44	90.00	(80.00, 90.00)	90.00	(80.00, 90.00)	80.00	(80.00, 90.00)	90.00	(80.00, 90.00)
	45-54	80.00	(80.00, 90.00)	80.00	(70.00, 90.00)	80.00	(70.00, 90.00)	80.00	(70.00, 90.00)
	55-64	80.00	(70.00, 90.00)	80.00	(70.00, 85.00)	80.00	(70.00, 80.00)	80.00	(70.00, 86.00)
	65-74	80.00	(70.00, 85.00)	70.00	(60.00, 80.00)	70.00	(60.00, 80.00)	75.00	(60.00, 80.00)
	75+	70.00	(60.00, 80.00)	70.00	(60.00, 80.00)	70.00	(60.00, 80.00)	70.00	(60.00, 80.00)
**Rural**									
All	Total	85.00	(80.00, 90.00)	80.00	(70.00, 90.00)	80.00	(70.00, 90.00)	80.00	(75.00, 90.00)
	15-24	90.00	(90.00, 100.00)	90.00	(90.00, 100.00)	90.00	(90.00, 95.00)	90.00	(90.00, 95.00)
	25-34	90.00	(90.00, 95.00)	90.00	(85.00, 95.00)	90.00	(80.00, 90.00)	90.00	(85.00, 90.00)
	35-44	90.00	(80.00, 90.00)	90.00	(80.00, 90.00)	90.00	(80.00, 90.00)	90.00	(80.00, 90.00)
	45-54	90.00	(80.00, 90.00)	80.00	(80.00, 90.00)	80.00	(78.00, 90.00)	80.00	(80.00, 90.00)
	55-64	80.00	(70.00, 90.00)	80.00	(70.00, 90.00)	80.00	(70.00, 90.00)	80.00	(70.00, 90.00)
	65-74	80.00	(70.00, 80.00)	70.00	(60.00, 80.00)	70.00	(60.00, 80.00)	75.00	(60.00, 80.00)
	75+	70.00	(60.00, 80.00)	70.00	(60.00, 80.00)	70.00	(60.00, 80.00)	70.00	(60.00, 80.00)
Males	Total	90.00	(80.00, 90.00)	80.00	(75.00, 90.00)	85.00	(80.00, 90.00)	85.00	(80.00, 90.00)
	15-24	90.00	(90.00, 100.00)	90.00	(90.00, 100.00)	90.00	(90.00, 95.00)	90.00	(90.00, 100.00)
	25-34	90.00	(90.00, 95.00)	90.00	(85.00, 95.00)	90.00	(85.00, 90.00)	90.00	(85.00, 95.00)
	35-44	90.00	(80.00, 90.00)	90.00	(80.00, 90.00)	90.00	(80.00, 90.00)	90.00	(80.00, 90.00)
	45-54	90.00	(80.00, 90.00)	85.00	(80.00, 90.00)	85.00	(80.00, 90.00)	85.00	(80.00, 90.00)
	55-64	80.00	(70.00, 90.00)	80.00	(70.00, 90.00)	80.00	(70.00, 90.00)	80.00	(70.00, 90.00)
	65-74	80.00	(70.00, 85.00)	80.00	(60.00, 80.00)	70.00	(60.00, 80.00)	80.00	(70.00, 80.00)
	75+	70.00	(60.00, 80.00)	70.00	(60.00, 80.00)	70.00	(60.00, 80.00)	70.00	(60.00, 80.00)
Females	Total	80.00	(80.00, 90.00)	80.00	(70.00, 90.00)	80.00	(70.00, 90.00)	80.00	(70.00, 90.00)
	15-24	90.00	(90.00, 100.00)	90.00	(90.00, 100.00)	90.00	(89.00, 95.00)	90.00	(90.00, 95.00)
	25-34	90.00	(85.00, 95.00)	90.00	(80.00, 94.00)	90.00	(80.00, 90.00)	90.00	(80.00, 90.00)
	35-44	90.00	(80.00, 90.00)	90.00	(80.00, 90.00)	90.00	(80.00, 90.00)	90.00	(80.00, 90.00)
	45-54	85.00	(80.00, 90.00)	80.00	(78.00, 90.00)	80.00	(70.00, 90.00)	80.00	(80.00, 90.00)
	55-64	80.00	(70.00, 90.00)	80.00	(70.00, 85.00)	80.00	(70.00, 85.00)	80.00	(70.00, 90.00)
	65-74	80.00	(70.00, 80.00)	70.00	(60.00, 80.00)	70.00	(60.00, 80.00)	70.00	(60.00, 80.00)
	75+	70.00	(60.00, 80.00)	70.00	(60.00, 80.00)	70.00	(60.00, 80.00)	70.00	(60.00, 80.00)

Table S5 Utility index scores (Mean, SD) of respondents by gender and age

		Urban								Rural								Total							
		Eastern		Central		Western		Total		Eastern		Central		Western		Total		Eastern		Central		Western		Total	
		Mean	SD	Mean	SD	Mean	SD	Mean	SD	Mean	SD	Mean	SD	Mean	SD	Mean	SD	Mean	SD	Mean	SD	Mean	SD	Mean	SD
All	Total	0.987	0.052	0.986	0.055	0.983	0.057	0.985	0.055	0.986	0.056	0.983	0.060	0.984	0.057	0.984	0.058	0.986	0.054	0.984	0.058	0.984	0.057	0.985	0.056
	15-24	0.999	0.011	0.998	0.025	0.998	0.013	0.998	0.017	0.999	0.021	0.998	0.019	0.997	0.034	0.998	0.027	0.999	0.016	0.998	0.022	0.998	0.027	0.998	0.023
	25-34	0.998	0.019	0.998	0.020	0.997	0.024	0.998	0.021	0.998	0.017	0.997	0.028	0.997	0.032	0.997	0.026	0.998	0.018	0.997	0.023	0.997	0.028	0.997	0.024
	35-44	0.996	0.023	0.994	0.032	0.994	0.031	0.994	0.029	0.995	0.031	0.994	0.034	0.995	0.031	0.995	0.032	0.995	0.027	0.994	0.033	0.994	0.031	0.995	0.031
	45-54	0.992	0.037	0.990	0.041	0.989	0.041	0.990	0.040	0.992	0.038	0.988	0.051	0.990	0.041	0.990	0.043	0.992	0.038	0.989	0.046	0.989	0.041	0.990	0.042
	55-64	0.987	0.048	0.985	0.053	0.981	0.056	0.984	0.052	0.985	0.054	0.979	0.062	0.978	0.062	0.981	0.059	0.986	0.051	0.981	0.058	0.980	0.059	0.983	0.056
	65-74	0.978	0.065	0.972	0.074	0.963	0.083	0.971	0.074	0.971	0.076	0.964	0.082	0.960	0.083	0.965	0.081	0.975	0.070	0.969	0.078	0.961	0.083	0.969	0.077
	75+	0.939	0.116	0.941	0.118	0.930	0.118	0.937	0.117	0.932	0.123	0.927	0.122	0.922	0.121	0.928	0.122	0.936	0.119	0.935	0.120	0.927	0.119	0.933	0.119
Males	Total	0.988	0.052	0.986	0.057	0.984	0.057	0.986	0.055	0.987	0.056	0.983	0.063	0.986	0.054	0.985	0.057	0.987	0.054	0.984	0.060	0.985	0.055	0.986	0.056
	15-24	0.999	0.012	0.997	0.035	0.999	0.013	0.998	0.021	0.999	0.027	0.998	0.017	0.996	0.045	0.997	0.035	0.999	0.020	0.998	0.028	0.997	0.036	0.998	0.029
	25-34	0.998	0.019	0.997	0.027	0.997	0.024	0.997	0.023	0.997	0.021	0.996	0.037	0.997	0.034	0.997	0.031	0.998	0.020	0.996	0.032	0.997	0.030	0.997	0.027
	35-44	0.996	0.024	0.994	0.036	0.993	0.036	0.994	0.033	0.994	0.036	0.993	0.043	0.995	0.033	0.994	0.037	0.995	0.031	0.993	0.040	0.994	0.034	0.994	0.035
	45-54	0.992	0.043	0.989	0.046	0.990	0.040	0.990	0.043	0.992	0.043	0.987	0.057	0.991	0.035	0.990	0.045	0.992	0.043	0.988	0.052	0.991	0.037	0.990	0.044
	55-64	0.988	0.051	0.984	0.062	0.984	0.047	0.985	0.054	0.986	0.054	0.980	0.062	0.982	0.055	0.983	0.057	0.987	0.052	0.982	0.062	0.983	0.052	0.984	0.055
	65-74	0.980	0.066	0.975	0.073	0.966	0.081	0.974	0.073	0.975	0.070	0.968	0.081	0.965	0.078	0.970	0.077	0.978	0.068	0.972	0.077	0.965	0.079	0.972	0.075
	75+	0.948	0.109	0.951	0.100	0.933	0.123	0.944	0.111	0.940	0.117	0.937	0.114	0.930	0.117	0.936	0.116	0.944	0.113	0.945	0.107	0.932	0.120	0.941	0.113
Females	Total	0.986	0.053	0.986	0.054	0.983	0.057	0.985	0.055	0.985	0.056	0.982	0.058	0.983	0.059	0.984	0.058	0.986	0.054	0.984	0.056	0.983	0.058	0.984	0.056
	15-24	0.999	0.011	0.999	0.010	0.998	0.014	0.999	0.012	0.999	0.013	0.998	0.020	0.999	0.015	0.998	0.016	0.999	0.012	0.999	0.016	0.998	0.014	0.999	0.014
	25-34	0.998	0.019	0.998	0.011	0.997	0.024	0.998	0.019	0.998	0.011	0.998	0.016	0.997	0.030	0.998	0.021	0.998	0.016	0.998	0.013	0.997	0.027	0.998	0.020
	35-44	0.996	0.022	0.994	0.028	0.994	0.026	0.995	0.025	0.996	0.025	0.995	0.024	0.994	0.029	0.995	0.026	0.996	0.024	0.995	0.026	0.994	0.027	0.995	0.026
	45-54	0.992	0.030	0.991	0.037	0.988	0.042	0.990	0.037	0.993	0.033	0.988	0.045	0.988	0.046	0.990	0.042	0.992	0.032	0.990	0.041	0.988	0.044	0.990	0.039
	55-64	0.986	0.046	0.985	0.044	0.978	0.062	0.984	0.051	0.983	0.055	0.978	0.062	0.975	0.068	0.979	0.062	0.985	0.051	0.981	0.054	0.976	0.065	0.981	0.057
	65-74	0.976	0.063	0.970	0.075	0.960	0.085	0.969	0.075	0.967	0.081	0.959	0.083	0.955	0.089	0.961	0.085	0.972	0.072	0.966	0.079	0.958	0.087	0.965	0.079
	75+	0.932	0.120	0.931	0.133	0.927	0.114	0.930	0.122	0.925	0.128	0.917	0.129	0.915	0.124	0.920	0.127	0.929	0.123	0.925	0.132	0.922	0.118	0.926	0.124

Table S6 Utility index scores (Median and Interquartile Range IQR) of respondents by gender and age

		Eastern		Central		Western		Total	
		Mean	IQR (Q1, Q3)	Mean	IQR (Q1, Q3)	Mean	IQR (Q1, Q3)	Mean	IQR (Q1, Q3)
**Urban & rural**									
All	Total	1.000	(1.000, 1.000)	1.000	(1.000, 1.000)	1.000	(1.000, 1.000)	1.000	(1.000, 1.000)
	15-24	1.000	(1.000, 1.000)	1.000	(1.000, 1.000)	1.000	(1.000, 1.000)	1.000	(1.000, 1.000)
	25-34	1.000	(1.000, 1.000)	1.000	(1.000, 1.000)	1.000	(1.000, 1.000)	1.000	(1.000, 1.000)
	35-44	1.000	(1.000, 1.000)	1.000	(1.000, 1.000)	1.000	(1.000, 1.000)	1.000	(1.000, 1.000)
	45-54	1.000	(1.000, 1.000)	1.000	(1.000, 1.000)	1.000	(1.000, 1.000)	1.000	(1.000, 1.000)
	55-64	1.000	(1.000, 1.000)	1.000	(1.000, 1.000)	1.000	(1.000, 1.000)	1.000	(1.000, 1.000)
	65-74	1.000	(0.973, 1.000)	1.000	(0.973, 1.000)	1.000	(0.973, 1.000)	1.000	(0.973, 1.000)
	75+	1.000	(0.896, 1.000)	1.000	(0.896, 1.000)	0.973	(0.886, 1.000)	1.000	(0.896, 1.000)
Males	Total	1.000	(1.000, 1.000)	1.000	(1.000, 1.000)	1.000	(1.000, 1.000)	1.000	(1.000, 1.000)
	15-24	1.000	(1.000, 1.000)	1.000	(1.000, 1.000)	1.000	(1.000, 1.000)	1.000	(1.000, 1.000)
	25-34	1.000	(1.000, 1.000)	1.000	(1.000, 1.000)	1.000	(1.000, 1.000)	1.000	(1.000, 1.000)
	35-44	1.000	(1.000, 1.000)	1.000	(1.000, 1.000)	1.000	(1.000, 1.000)	1.000	(1.000, 1.000)
	45-54	1.000	(1.000, 1.000)	1.000	(1.000, 1.000)	1.000	(1.000, 1.000)	1.000	(1.000, 1.000)
	55-64	1.000	(1.000, 1.000)	1.000	(1.000, 1.000)	1.000	(1.000, 1.000)	1.000	(1.000, 1.000)
	65-74	1.000	(1.000, 1.000)	1.000	(0.973, 1.000)	1.000	(0.973, 1.000)	1.000	(0.973, 1.000)
	75+	1.000	(0.923, 1.000)	1.000	(0.923, 1.000)	1.000	(0.892, 1.000)	1.000	(0.923, 1.000)
Females	Total	1.000	(1.000, 1.000)	1.000	(1.000, 1.000)	1.000	(1.000, 1.000)	1.000	(1.000, 1.000)
	15-24	1.000	(1.000, 1.000)	1.000	(1.000, 1.000)	1.000	(1.000, 1.000)	1.000	(1.000, 1.000)
	25-34	1.000	(1.000, 1.000)	1.000	(1.000, 1.000)	1.000	(1.000, 1.000)	1.000	(1.000, 1.000)
	35-44	1.000	(1.000, 1.000)	1.000	(1.000, 1.000)	1.000	(1.000, 1.000)	1.000	(1.000, 1.000)
	45-54	1.000	(1.000, 1.000)	1.000	(1.000, 1.000)	1.000	(1.000, 1.000)	1.000	(1.000, 1.000)
	55-64	1.000	(1.000, 1.000)	1.000	(1.000, 1.000)	1.000	(0.973, 1.000)	1.000	(1.000, 1.000)
	65-74	1.000	(0.973, 1.000)	1.000	(0.973, 1.000)	1.000	(0.963, 1.000)	1.000	(0.973, 1.000)
	75+	1.000	(0.896, 1.000)	1.000	(0.886, 1.000)	0.973	(0.859, 1.000)	0.973	(0.886, 1.000)
**Urban**									
All	Total	1.000	(1.000, 1.000)	1.000	(1.000, 1.000)	1.000	(1.000, 1.000)	1.000	(1.000, 1.000)
	15-24	1.000	(1.000, 1.000)	1.000	(1.000, 1.000)	1.000	(1.000, 1.000)	1.000	(1.000, 1.000)
	25-34	1.000	(1.000, 1.000)	1.000	(1.000, 1.000)	1.000	(1.000, 1.000)	1.000	(1.000, 1.000)
	35-44	1.000	(1.000, 1.000)	1.000	(1.000, 1.000)	1.000	(1.000, 1.000)	1.000	(1.000, 1.000)
	45-54	1.000	(1.000, 1.000)	1.000	(1.000, 1.000)	1.000	(1.000, 1.000)	1.000	(1.000, 1.000)
	55-64	1.000	(1.000, 1.000)	1.000	(1.000, 1.000)	1.000	(1.000, 1.000)	1.000	(1.000, 1.000)
	65-74	1.000	(1.000, 1.000)	1.000	(0.973, 1.000)	1.000	(0.973, 1.000)	1.000	(0.973, 1.000)
	75+	1.000	(0.896, 1.000)	1.000	(0.923, 1.000)	1.000	(0.886, 1.000)	1.000	(0.896, 1.000)
Males	Total	1.000	(1.000, 1.000)	1.000	(1.000, 1.000)	1.000	(1.000, 1.000)	1.000	(1.000, 1.000)
	15-24	1.000	(1.000, 1.000)	1.000	(1.000, 1.000)	1.000	(1.000, 1.000)	1.000	(1.000, 1.000)
	25-34	1.000	(1.000, 1.000)	1.000	(1.000, 1.000)	1.000	(1.000, 1.000)	1.000	(1.000, 1.000)
	35-44	1.000	(1.000, 1.000)	1.000	(1.000, 1.000)	1.000	(1.000, 1.000)	1.000	(1.000, 1.000)
	45-54	1.000	(1.000, 1.000)	1.000	(1.000, 1.000)	1.000	(1.000, 1.000)	1.000	(1.000, 1.000)
	55-64	1.000	(1.000, 1.000)	1.000	(1.000, 1.000)	1.000	(1.000, 1.000)	1.000	(1.000, 1.000)
	65-74	1.000	(1.000, 1.000)	1.000	(0.973, 1.000)	1.000	(0.973, 1.000)	1.000	(0.973, 1.000)
	75+	1.000	(0.923, 1.000)	1.000	(0.937, 1.000)	1.000	(0.896, 1.000)	1.000	(0.923, 1.000)
Females	Total	1.000	(1.000, 1.000)	1.000	(1.000, 1.000)	1.000	(1.000, 1.000)	1.000	(1.000, 1.000)

	15-24	1.000	(1.000, 1.000)	1.000	(1.000, 1.000)	1.000	(1.000, 1.000)	1.000	(1.000, 1.000)
	25-34	1.000	(1.000, 1.000)	1.000	(1.000, 1.000)	1.000	(1.000, 1.000)	1.000	(1.000, 1.000)
	35-44	1.000	(1.000, 1.000)	1.000	(1.000, 1.000)	1.000	(1.000, 1.000)	1.000	(1.000, 1.000)
	45-54	1.000	(1.000, 1.000)	1.000	(1.000, 1.000)	1.000	(1.000, 1.000)	1.000	(1.000, 1.000)
	55-64	1.000	(1.000, 1.000)	1.000	(1.000, 1.000)	1.000	(0.973, 1.000)	1.000	(1.000, 1.000)
	65-74	1.000	(0.973, 1.000)	1.000	(0.973, 1.000)	1.000	(0.964, 1.000)	1.000	(0.973, 1.000)
	75+	1.000	(0.896, 1.000)	1.000	(0.896, 1.000)	0.973	(0.886, 1.000)	1.000	(0.896, 1.000)
**Rural**									
All	Total	1.000	(1.000, 1.000)	1.000	(1.000, 1.000)	1.000	(1.000, 1.000)	1.000	(1.000, 1.000)
	15-24	1.000	(1.000, 1.000)	1.000	(1.000, 1.000)	1.000	(1.000, 1.000)	1.000	(1.000, 1.000)
	25-34	1.000	(1.000, 1.000)	1.000	(1.000, 1.000)	1.000	(1.000, 1.000)	1.000	(1.000, 1.000)
	35-44	1.000	(1.000, 1.000)	1.000	(1.000, 1.000)	1.000	(1.000, 1.000)	1.000	(1.000, 1.000)
	45-54	1.000	(1.000, 1.000)	1.000	(1.000, 1.000)	1.000	(1.000, 1.000)	1.000	(1.000, 1.000)
	55-64	1.000	(1.000, 1.000)	1.000	(1.000, 1.000)	1.000	(1.000, 1.000)	1.000	(1.000, 1.000)
	65-74	1.000	(0.973, 1.000)	1.000	(0.973, 1.000)	1.000	(0.964, 1.000)	1.000	(0.973, 1.000)
	75+	1.000	(0.896, 1.000)	1.000	(0.886, 1.000)	0.973	(0.86, 1.000)	1.000	(0.886, 1.000)
Males	Total	1.000	(1.000, 1.000)	1.000	(1.000, 1.000)	1.000	(1.000, 1.000)	1.000	(1.000, 1.000)
	15-24	1.000	(1.000, 1.000)	1.000	(1.000, 1.000)	1.000	(1.000, 1.000)	1.000	(1.000, 1.000)
	25-34	1.000	(1.000, 1.000)	1.000	(1.000, 1.000)	1.000	(1.000, 1.000)	1.000	(1.000, 1.000)
	35-44	1.000	(1.000, 1.000)	1.000	(1.000, 1.000)	1.000	(1.000, 1.000)	1.000	(1.000, 1.000)
	45-54	1.000	(1.000, 1.000)	1.000	(1.000, 1.000)	1.000	(1.000, 1.000)	1.000	(1.000, 1.000)
	55-64	1.000	(1.000, 1.000)	1.000	(1.000, 1.000)	1.000	(1.000, 1.000)	1.000	(1.000, 1.000)
	65-74	1.000	(1.000, 1.000)	1.000	(0.973, 1.000)	1.000	(0.973, 1.000)	1.000	(0.973, 1.000)
	75+	1.000	(0.923, 1.000)	1.000	(0.896, 1.000)	1.000	(0.888, 1.000)	1.000	(0.896, 1.000)
Females	Total	1.000	(1.000, 1.000)	1.000	(1.000, 1.000)	1.000	(1.000, 1.000)	1.000	(1.000, 1.000)
	15-24	1.000	(1.000, 1.000)	1.000	(1.000, 1.000)	1.000	(1.000, 1.000)	1.000	(1.000, 1.000)
	25-34	1.000	(1.000, 1.000)	1.000	(1.000, 1.000)	1.000	(1.000, 1.000)	1.000	(1.000, 1.000)
	35-44	1.000	(1.000, 1.000)	1.000	(1.000, 1.000)	1.000	(1.000, 1.000)	1.000	(1.000, 1.000)
	45-54	1.000	(1.000, 1.000)	1.000	(1.000, 1.000)	1.000	(1.000, 1.000)	1.000	(1.000, 1.000)
	55-64	1.000	(1.000, 1.000)	1.000	(0.973, 1.000)	1.000	(0.973, 1.000)	1.000	(1.000, 1.000)
	65-74	1.000	(0.973, 1.000)	1.000	(0.968, 1.000)	1.000	(0.937, 1.000)	1.000	(0.973, 1.000)
	75+	0.973	(0.886, 1.000)	0.973	(0.859, 1.000)	0.973	(0.851, 1.000)	0.973	(0.860, 1.000)

Table S7 Percentage of respondents reporting moderate or severe problems in different countries

Country	Data collection year	Simple size	Mobility	Self-care	Usual activities	Pain/ discomfort	Anxiety/ depression	Utility index	VAS score
Argentina	2005	41392	10.8	2.9†	8	30.9	22.8	0.902	75.30
Australia	2011	5555	16.4	3.1	15.3	40.1	16.3	0.910	78.55
Belgium	2001–2003	2,411	12.6	4	12.4	28.5	6.6	0.891	77.60
China	2010	8,031	5.1	2.8	5.2	10.7	8.7	0.951	80.40
Denmark	2000–2001	16861	10.7	2.5	17.9	36.7	16.1	0.887	83.70
England	2008	14763	19.4	5.6	17	35.3	19.3	0.858	—
Finland	2000	8028	26.3	8.6	21	47.8	13.9	0.815	—
France	2001–2003	2892	13.4	4	10	35.9	15	0.892	76.80
Germany	2001–2003	3552	15.9	2.7	9.9	27.6	4.3	0.938	77.30
Greece	1998	464	13.3	5.7	10.5	16.8	10.7	0.913	79.00
Hungary	2000	5503	19.6	6.5	14.8	39.2	35.2	0.823	71.10
Italy	2013	6800	13.5	3.9	12	41.6	33.5	0.915	78.22
Japan	2013	1143	9.3	3.2	9.9	25	12.4	0.909	—
Korea	2007	1307	5.9	0.8	4.1	21.3	17.4	0.958	79.50
Netherlands	2001–2003	2367	11.5	3.4	13.5	34.2	3.5	0.910	82.00
New Zealand	1999	1327	20	4.4	21.5	40.8	21.2	0.848	80.80
Poland	2014	3941	23.3	9.4	19.2	45.8	33.4	0.893	73.70
Portuguese	2014	1500	16.7	4.8	16.3	44.7	34.4	0.758	74.90
Singapore	2009-2010	6166	3.6	0.5	2.2	15.4	8.2	0.950	—
Slovenia	2000	742	29.8	14	32.9	47.2	36.4	0.788	76.40
Spain	2001–2003	5473	13.7	4.1	11.7	22.9	7.8	0.929	75.00
Sri Lanka	2012-2013	780	18.6	6	7.8	33.4	15.4	0.850	81.00
Sweden	1994	534	8.6	1.5	7.9	40.8	26	0.851	83.30
Switzerland	2007	1956	7.9	2.4	8.8	45.7	31.6	0.830	81.70
Thailand	2007	1409	26.3	8.7	22.7	65	47.4	0.742	79.40
United Kingdom	1993	3395	18.4	4.3	16.3	33	21	0.856	82.80
United States	2000–2002	38678	18.5	3.7	17.9	48.3	23.2	0.867	80.00
**China**	**2013**	**188720**	**5.86**	**3.05**	**4.64**	**12.61**	**5.27**	**0.985**	**80.90**

†Note: Figures in red indicate reported problems lower than those in China; figures in blue indicate Utility index or VAS score higher than those in China.
